# Fractal dimension based geographical clustering of COVID-19 time series data

**DOI:** 10.1038/s41598-023-30948-7

**Published:** 2023-03-15

**Authors:** Yessika Adelwin Natalia, Christel Faes, Thomas Neyens, Pieter Chys, Naïma Hammami, Geert Molenberghs

**Affiliations:** 1 I-BioStat, Data Science Institute, Hasselt University, 3500 Hasselt, Belgium; 2 I-BioStat, KU Leuven, 3000 Leuven, Belgium; 3 Team Infection Prevention and Vaccination, Agency for Care and Health, 1030 Brussels, Belgium

**Keywords:** Infectious diseases, Machine learning, Statistics, Epidemiology

## Abstract

Understanding the local dynamics of COVID-19 transmission calls for an approach that characterizes the incidence curve in a small geographical unit. Given that incidence curves exhibit considerable day-to-day variation, the fractal structure of the time series dynamics is investigated for the Flanders and Brussels Regions of Belgium. For each statistical sector, the smallest administrative geographical entity in Belgium, fractal dimensions of COVID-19 incidence rates, based on rolling time spans of 7, 14, and 21 days were estimated using four different estimators: box-count, Hall-Wood, variogram, and madogram. We found varying patterns of fractal dimensions across time and location. The fractal dimension is further summarized by its mean, variance, and autocorrelation over time. These summary statistics are then used to cluster regions with different incidence rate patterns using *k*-means clustering. Fractal dimension analysis of COVID-19 incidence thus offers important insight into the past, current, and arguably future evolution of an infectious disease outbreak.

## Introduction

More than three years after the first outbreak of coronavirus disease 2019 (COVID-19) in Wuhan, China^1^, the world is still on high alert due to this pandemic. The ongoing transmission has been a major concern in most countries, including Belgium, which was hit by multiple waves of COVID-19 cases. As reported by Sciensano, the Belgian institute for public health, the first wave particularly hit the elderly population in March-April 2020^2^. The second wave hit harder in the younger population from September 2020 until January 2021, with the second wave generally more severe, against the background of the very limited testing capacity in the Spring of 2020. Over the period January–June 2020, only 61,984 infections were test confirmed, with 588,056 infections test confirmed over the period July–December 2020. Over the same periods, COVID-19 related hospitalisations were 18,071 and 31,400, respectively. Peak ICU occupancy was 1286 on 8 April 2020 and 1474 on 9 November 2020, respectively. COVID-19 mortality was 9736 in the first half and 10,110 in the second half of 2020.

Multiple variants have been reported globally, some of them declared to be variants of concern (VOC) by the World Health Organization (WHO). The Alpha variant started circulating in December 2020, while Beta and Gamma followed early in 2021. The Delta variant circulated from May 2021 to early 2022, and the Omicron variant took off in late November 2021^3^. Rapidly changing dynamics of COVID-19 transmission urged researchers to model the epidemic curve and predict the pattern of this disease. Mathematical compartmental models to describe and predict these changes^4–6^ have frequently been used, while other studies use statistical models to assess trends in the disease, e.g., time series analysis^7,8^ or spatio-temporal modeling^9–12^.

Daily or weekly incidence data is often subject to considerable heterogeneity, particularly in the field of infectious diseases. In a relatively stable period, we would see little changes in the incidences. However, during an epidemic period, we could observe different patterns of incidences. On top of this, the amount of heterogeneity increases as the study region becomes smaller. Since geographical scaling comes into play, it is sensible to consider the incidence curve as a fractal structure. A fractal is a self-similar structure at different scales^13^. The basic theory of fractal structures was first introduced by Mandelbrot and offers an elegant mathematical paradigm to describe a variety of complex real-world objects^14^. An important aspect of fractal theory is the fractal dimension of an object^15^. It can be used to characterize the geometric complexity of an object. In the past, the concept of fractal dimension was mainly used in geography, mathematics, or engineering^16–19^. Recently, however, the use of fractal dimension has been expanded to diverse scientific fields, such as economy and medicine^20–22^. Based on the notion that very noisy information can also carry a signal, we can examine the fractal dimension to evaluate the epidemic curve. A study from Păcurar and Necula showed that a fractal point of view can be useful to find new information of different outbreaks or predictions^23^.

A time-series curve is an object of which the complexity could be described by fractal dimension. Therefore, we aimed to evaluate the patterns of COVID-19 cases in multiple areas in Belgium from a fractal-dimension perspective. We hypothesize that areas with complex incidence patterns will have higher fractal dimension estimates and vice versa. Based on the statistical characteristics depicted by fractal dimension estimation, we could then cluster areas with similar epidemic patterns.

## Methods

### Simulation data

To obtain extra background information on our proposed statistical analysis, we conducted first a simulation study. We simulated possible daily COVID-19 incidence case data at a very fine geographical level using a time series model. For simplicity, we used white noise from an autoregressive integrated moving average (ARIMA) model, defined as:

1 $$\begin{aligned} y_{t}=\mu +\omega _{t}, \end{aligned}$$

where $y_{t}$ is the number of cases at a certain time point, $\mu$ is a constant, and error term $\omega _{t} \sim Po(\lambda )$. Different values of the $\lambda$ parameter in the Poisson distribution were used to increase the complexity of the daily incidence rate curve.

Note that the $\lambda$ parameter is used to model the error term in the white noise model and is a fixed value. The error term is indeed time-dependent but the $\lambda$ value is constant. We assume that we will observe more cases in a day within a certain period as the transmission increases and the incidence curve will appear to be more complex than in other periods.

### Real world data

Individual data of daily COVID-19 confirmed cases at the level of statistical sector were retrieved via ZorgAtlas platform managed by the Agency for Care and Health (https://www.zorg-en-gezondheid.be/). Belgium is divided into 3 geographical Regions: Flanders, Brussels, and Walloon. The agency is responsible to collect data in the Flanders Region, but also integrates data from the Brussels Region. Consequently, our work focuses on these two geographical entities.

**Figure 1:**
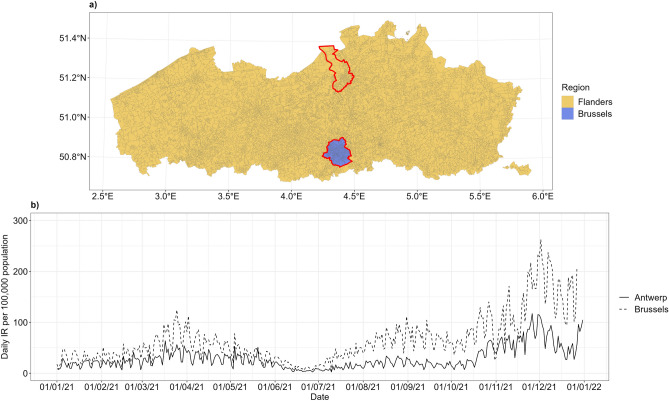
Statistical sectors in Flanders and Brussels Region. The location of selected study areas is marked by red line in a). Daily incidence rates per 100,000 inhabitants of these two areas are shown in b). The map is adapted from https://statbel.fgov.be/en/open-data/statistical-sectors-2020 using R 4.2.1 (https://CRAN.R-project.org/).

Each region is subdivided into provinces, which in turn consist of multiple municipalities. Each municipality is further subdivided into statistical sectors, based on structural characteristics of social, economic, urban planning, and/or morphological nature^24^. In 2020, Belgium consisted of 19,794 statistical sectors: 9194 were located in the Flanders region, 724 in the Brussels region (Fig. 1a). The statistical sectors map is made available online by Statbel, the Belgian national statistics institute^25^. The population data were also made available by this institute. In 2020, Belgium had a population of 11,492,641 inhabitants with 6,626,475 inhabitants in Flanders Region and 1,215,012 inhabitants in Brussels Region.

Due to the large number of statistical sectors in our data, we confine our attention to the Antwerp municipality (Fig. 1a, red line on the top) as well as the Brussels Region (Fig. 1a, red line on the bottom). Antwerp is located in the north of Flanders and consists of 299 statistical sectors, while Brussels is located in the center of Belgium and consists of 724 statistical sectors. In 2020, Antwerp had a population of 529,247 inhabitants and Brussels had a population of 1,215,012 inhabitants. These two areas represent a typical urban area in Belgium with middle socioeconomic status and dense population with different nationalities and cultural backgrounds^26,27^.

### Statistical analysis

The fractal dimension can be estimated using a range of techniques. Some methods have been compared and described in detail by Gneiting et al.^28^. Based on this study, we decided to use four basic methods to estimate the fractal dimension: the box-count, Hall-Wood, variogram, and madogram estimators. The basic characteristics of each method are summarized in Table 1.

**Table 1:** Characteristics of fractal dimension estimators.

Estimator	General description	Calculation formula	Advantages	Disadvantages
Box-count	Number of boxes at a certain scale required to cover the time series data at increasingly fine scales.	$\hat{\text{ D }}_{\text{ BC }}=\lim _{\delta \rightarrow 0}\frac{\log \, N_{\delta }}{-\log \, \delta }$	Simple and intuitive formulation^29^.	Lower estimation, increasing mean squared errors and asymptotic bias with increasing number of points in the regression^30–32^.
Hall-Wood	Modification of box-count estimator. The total area of boxes that cover the time series data is used instead of the number of boxes.	$\hat{\text{ D }}_{\text{ HW }}=2-\frac{\log \, \hat{A}(2/n)-\log \, \hat{A}(1/n)}{\log \, 2}$	Better accuracy compared to the box-count estimator^28^.	It can only be applied to a stationary Gaussian process with equally spaced points^32^.
Variogram & madogram	Quantification of variability between data points as a function of distance.	$\hat{\text{ D }}_{\text{ V;p }}=2-\frac{1}{p}\frac{\log \, \hat{V}_{p}(2/n)-\log \, \hat{V}_{p}(1/n)}{\log \, 2}$	More efficient than the Hall-Wood estimator. The madogram estimator is more robust against outliers^28^.	It can fail easily under some non-Gaussian processes, in particular for the variogram estimator^28^.

$$N = $$ number of boxes; $$\delta = $$ boxwidth; $$\hat{A}$$ = total area of of boxes; $$n = $$ number of observations; $$\hat{V} = $$ moments estimator; $$p = $$ order (2 for variogram, 1 for madogram)

Daily COVID-19 incidence rates were calculated for each statistical sector, based upon which we estimated the local fractal dimension that captures information about the evolution in daily incidence rate within a certain period. Fractal dimension estimation of a curve typically ranges from 1 to 2 with value 1 corresponding to a structure that is similar to a straight line and values close to 2 representing a more ‘space filling’ structure. Thus, a higher fractal dimension value indicates a higher complexity of the daily COVID-19 incidence curve. Due to possible short-term fluctuations in reporting efforts we defined sliding windows of width 7, 14, and 21 days for which the fractal dimensions were estimated. Their values were plotted to obtain a fractal dimension curve. Note for a given point in time, the depicted fractal dimension estimate is based on the sliding window period that ends on that particular date. The mean, variance, and autocorrelation of the fractal dimension curve were calculated per statistical sector and displayed by means of choropleth maps.

Qualitative combinations of mean, variance, and autocorrelation values can be used to characterize statistical sectors. However, Gneiting et al. reported that different methods lead to different fractal dimension estimates^28^.Different fractal dimension values would eventually impact the mean, variance, and autocorrelation values. Therefore, we opt for clustering the local fractal dimension statistics using *k*-means clustering because it is the simplest type of finite mixture models and computationally fast^33^. The optimal number of clusters was first determined using the elbow method, which is heuristic but simple to implement. This method optimizes the number of clusters based on the sum of squares of the Euclidean distances between each point and its corresponding centroid. The relationship between the sum of the square and the possible number of clusters *k* is plotted in a curve. The curve will be flattened out when the value of *k* increases and the optimal number of clusters lies in the highest curvature of elbow; i.e., adding another cluster will not give additional benefit to classifying the data^34^. The value of each centroid cluster was obtained and compared with the mean of local fractal dimension statistics. For each of these, the label ‘low’ indicates that the centroid values are lower than the mean of the respective statistic and the label ‘high’ indicates that the centroid values are higher than that.

The results in this paper were obtained using R 4.2.1 available from the Comprehensive R Archive Network (CRAN) at https://CRAN.R-project.org/. The fractal dimension was calculated using package fractaldim^28^.

### Ethics declaration

This study has been approved by the Agency for Care and Health (GE0-1GDF2IA-WT/1GD305/20073674). It was conducted in accordance with international ethical standards (Declaration of Helsinki 1964). It was conducted in accordance with the General Data Protection Regulation (GDPR) and a data processing agreement between the Agency for Care and Health and Hasselt University was concluded. Participant information was coded and held securely. De-identification was performed on data content to comply with the Data Protection Regulation scope.

## Results

### Simulation study

An example of the simulated curves and their fractal dimension are shown in Supplementary Figs. S1–S4 online. The daily incidence rate curve with a small $\lambda \in [0.01;0.11]$ mimics a mild to heavy sporadic transmission, while a curve with $\lambda \in [0.13;0.23]$ mimics mild community transmission. Higher $\lambda$ values mimic heavy community transmission. The fractal dimension curves calculated via each method were shown in the top part of each panel. We observe that different methods lead to different fractal dimension estimates, with the box-count estimator typically giving the lowest estimate. As expected, the fractal dimension curve of the 7-day sliding window showed more fluctuations as compared to sliding windows of 14 and 21 days. We then calculated the mean, variance, and autocorrelation values of these curves.

Supplementary Fig. S5–S6 show boxplots of the local fractal dimension statistics for each estimator and sliding window after 1,000 replications. We observe an increase in the mean fractal dimension value (top panels) with increasing $\lambda$ values. This indicates that a higher mean value represents higher complexity of the daily incidence rate curve, i.e., community transmission. The variance of the fractal dimension curves (middle panels) with $\lambda \in [0.01;0.23]$ is higher than those with higher $\lambda$ values. Low variance indicates little change in the local fractal dimension. The boxplots in the bottom panels show a high autocorrelation value for each $\lambda$ value for the variogram and madogram estimators, which suggests a high temporal correlation when the fractal dimension is calculated using these methods.

We further calculated the optimal number of clusters using the elbow method. For each estimator and sliding window, the highest curvature of the elbow lies at value $k = 4$. The curvature does not change much beyond this value, therefore we concluded that the optimal number of clusters was four. We then used this value to further classify each data point using *k*-means clustering. Supplementary Figs. S7–S10 give an example of the clusters detected for each estimator using different sliding windows. We could see a contrast between clusters of lower and higher $\lambda$ values. We compared the centroid values to the mean of local fractal dimension statistics as noted in the legends. To ease the interpretation, we summarized this classification as shown in Table 2.

**Table 2:** Proposed classification of local fractal dimension based on mean, variance, and autocorrelation value.

Mean	Variance	Autocorrelation	Possible transmission type
Low	Low	Low	Mild sporadic
Low	Low	High	Mild sporadic
Low	High	Low	Heavy sporadic
Low	High	High	Heavy sporadic
High	High	Low	Mild community
High	High	High	Mild community
High	Low	Low	Heavy community
High	Low	High	Heavy community

For comparison’s sake, we also analyzed our simulation data using another known method. Based on the simplicity and the availability of R packages for this context, we opted to use so-called dynamic time warping, via its implementation in the package dtwclust. This method utilizes the dynamic time warping distance as a dissimilarity measure to find the optimum warping path between two series under certain constraints^35^. The results showed that our method can detect clusters with similar features as dynamic time warping, i.e., incidence rate curves with similar shapes are clustered together. The time required to complete an analysis is relatively short for both methods (2.7 seconds for local fractal dimension and 1.3 seconds for *k*-means dynamic time warping). An advantage of our method is that we can retrieve the characteristics of each cluster via the mean, variance, and autocorrelation values classification to directly compare different curves. An analog to this feature is not readily available for dynamic time warping, given that we have to manually compare each centroid curve to retrieve the characteristics of each cluster.

### Real world data analysis

#### COVID-19 incidence

We retrieved the daily COVID-19 cases from 1 July 2020 until 31 July 2021. There were 492,514 cases reported with known residential statistical sectors. The number of daily reported cases in Antwerp increased considerably in October 2020 and reached the highest peak in November 2020 (Fig. 1b). The numbers declined in December 2020 but remained relatively high until June 2021 when they declined slightly. The cases exhibited again an increasing trend in July 2021. A similar but somewhat less pronounced trend was observed in Brussels.

#### Local fractal dimension

**Figure 2:**
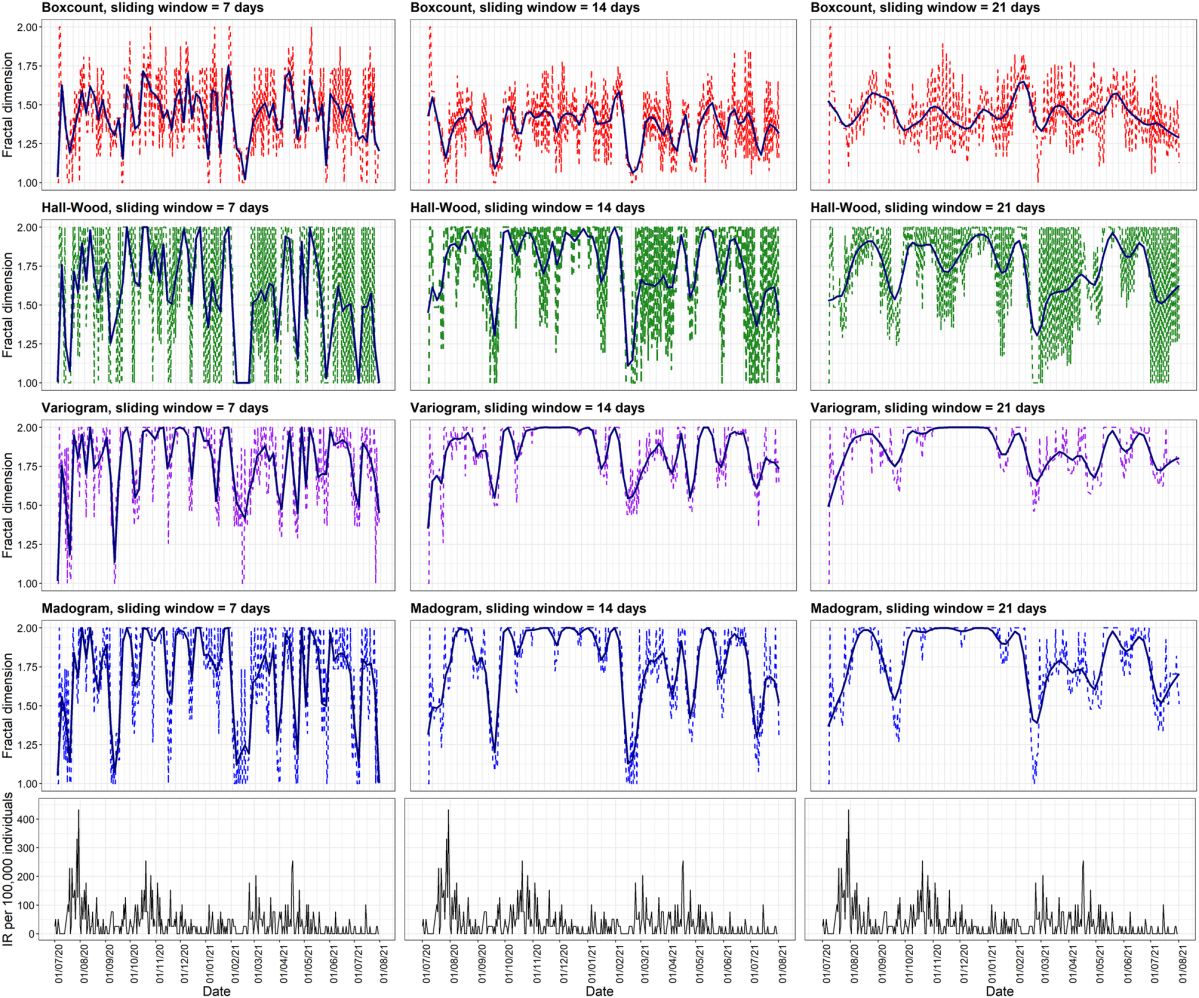
Local fractal dimension of COVID-19 incidence rate in *De Peperbus*, Antwerp. The raw fractal dimension value is depicted by the dashed line. Dark blue solid lines correspond to LOESS regression of the local fractal dimension.

**Figure 3:**
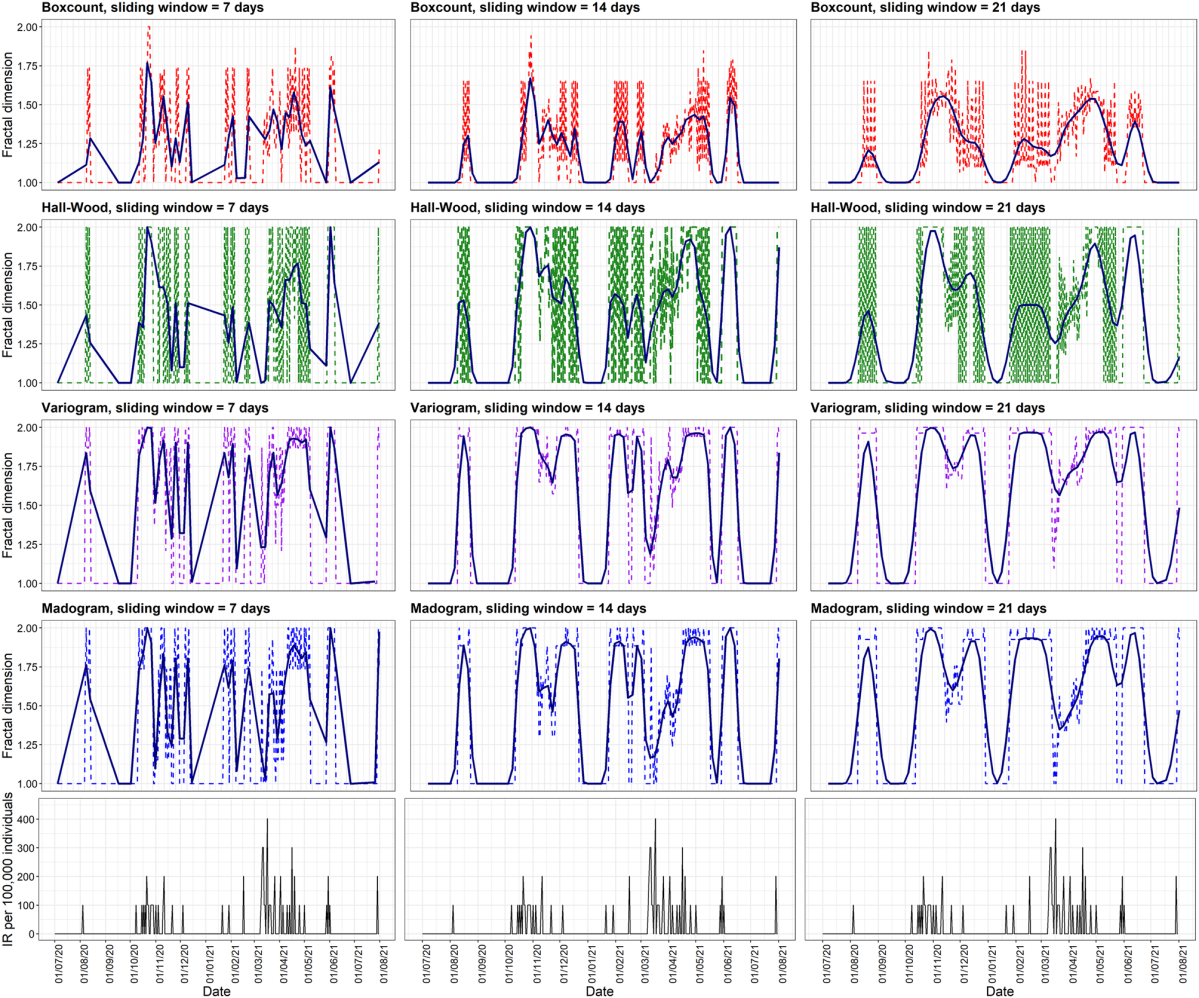
Local fractal dimension of COVID-19 incidence rate in *Prinshoeveland*, Antwerp. The raw fractal dimension value is depicted by the dashed line. Dark blue solid lines correspond to LOESS regression of the local fractal dimension.

To illustrate the local fractal dimension, we selected two statistical sectors as shown in Figs. 2 and 3. Figure 2 shows results for *De Peperbus*, in which COVID-19 cases have been continually reported since the beginning of July 2020 and with higher daily incidence rates in July and October 2020. In contrast to this sector, *Prinshoeveland*, had a lower incidence rate with a longer period of no reported cases (Fig. 3). A lower and less chaotic fractal dimension curve could be observed in *Prinshoeveland*. This suggests a lower complexity of the COVID-19 daily incidence curve in this sector. The incidence curve is presented by the black line at the bottom of each panel. The left, middle, and right panels correspond to analyses with moving windows of 7, 14, and 21 days, respectively. The colored lines in the rows correspond to, respectively, the box-count, Hall-Wood, variogram, and madogram methods.

One observes a large difference in case counts when comparing Figs. 2 and  3. While caution is needed when interpreting the results, thanks to the definition of scale-invariant fractal dimension, a comparison is still possible. That said, small case numbers will impact the statistical uncertainty with which fractal dimensions are investigated. Also, the different methods lead to different fractal dimensions, with the box-count estimator often yielding the lowest estimate. The local fractal dimension curve based on a 7-day sliding window showed more fluctuations as compared to sliding windows of 14 and 21 days. For the purpose of comparison, we smoothed the local fractal dimension curve using locally weighted regression (LOESS). Evidently, findings should always be scrutinized further and, as such, the proposed methodology can help characterize a statistical sector, but human interpretation, perhaps bringing in sector-specific but important background knowledge, will arguably always be needed.

#### Transmission classification

**Figure 4:**
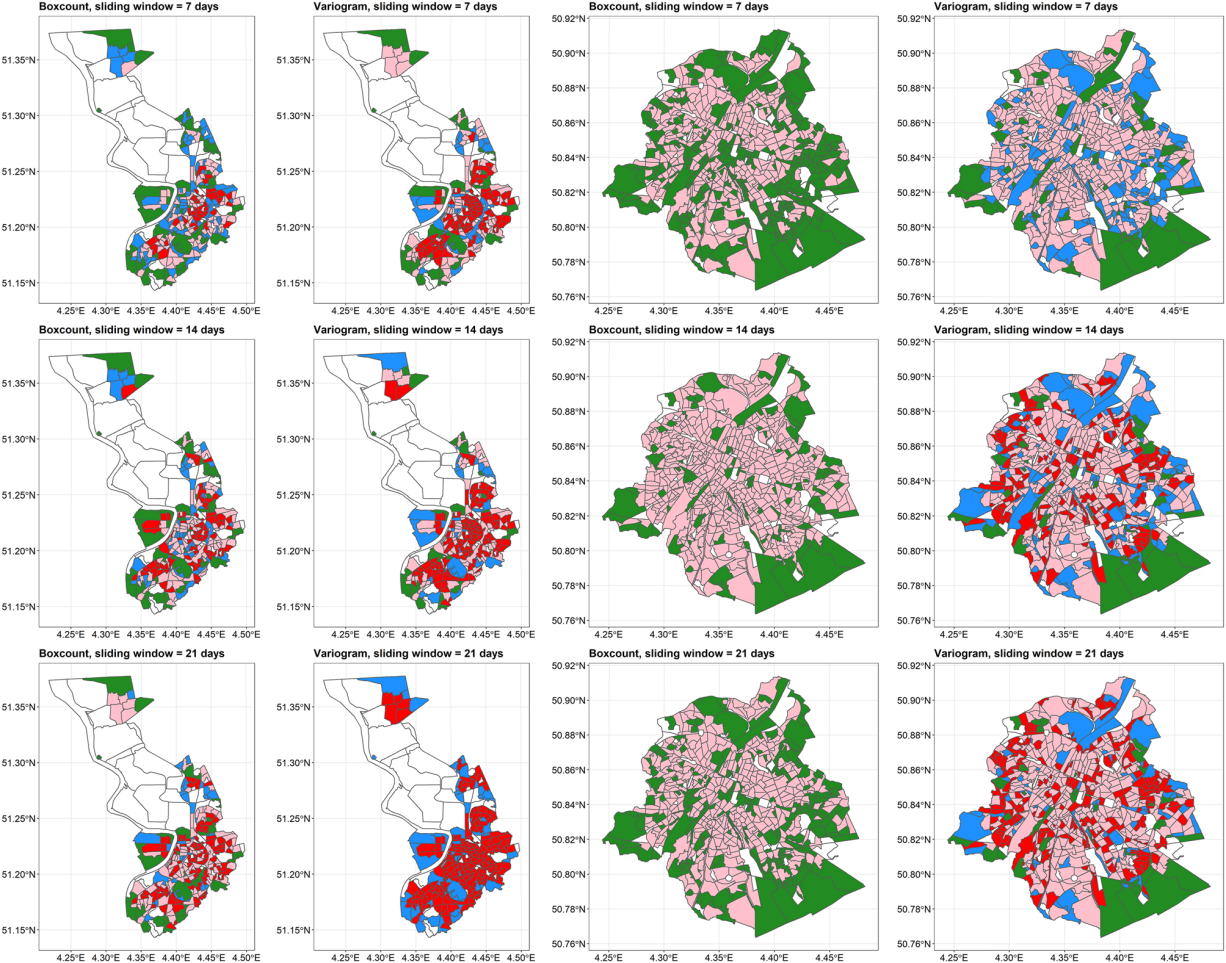
Classification of statistical sectors in Antwerp municipality (left panels) and Brussels Region (right panels) calculated by box-count and variogram estimator. The color green, blue, pink, and red correspond to mild sporadic, heavy sporadic, mild community, and heavy community, respectively. White color represents no reported cases. The map is adapted from https://statbel.fgov.be/en/open-data/statistical-sectors-2020 using R 4.2.1 (https://CRAN.R-project.org/).

For each statistical sector, we calculated the mean, variance, and autocorrelation of the fractal dimension curve. The raw values per statistical sector are shown in Supplementary Figs. S11–S18. These results were then compiled into choropleth maps based on the classification presented in Table 2. The classification based on box-count and variogram estimator is depicted in Fig. 4. The results based on other estimators are shown in Supplementary Fig. S19.

We found that the box-count estimator with a sliding window of 21 days could differentiate a similar number of clusters as the variogram estimator with a sliding window of 7 days. Similar results could be observed using the Hall-Wood and madogram estimators. Despite some differences, based on these results we could detect a cluster of mild to heavy community transmission around the city center and to some extent, the northern part of the Antwerp municipality. In the Brussels Region, the cluster of mild to heavy community transmission is distributed all over the region.

## Discussion

Our analyses have illustrated that structural elements of the fractal dimension curve derived from a statistical sector’s COVID-19 incidence curve can be used to characterize how the epidemic behaves in a given sector.

We retrieved COVID-19 data at the statistical sector level from July 2020 onward. However, data in some areas were not available until November 2020, particularly in the Brussels Region, which was included in the project later. This will increase the heterogeneity of the available time-series data. Fractal dimension analysis is particularly useful to simplify a dependency structure by using a few indices when we consider time series data as a fractal structure^36^. Castillo and Melin reported that the complex behavior of time series data could be measured and compared among different periods and countries to forecast the evolution and decide on possible non-pharmaceutical interventions based on the current situation^37^.

Different estimators yielded different estimates of the fractal dimension. In this study, the box-count estimator showed the lowest fractal dimension among all methods. Gneiting et al. compared different fractal dimension estimators and their study reported that the box-count estimator generally shows a downward bias^28^. They also reported that the variogram estimator has the lowest mean square error among other estimators and that the madogram estimator is more robust against outliers. We did not choose a specific estimator *a priori*. Based on the results of our study, we are inclined to prefer the variogram or madogram estimators.

Different statistical sectors have different patterns of their local fractal dimension. In the case of *De Peperbus*, most of the time we observed stable high signals, i.e., a high fractal dimension. This could be explained by the relatively chaotic pattern of COVID-19 daily incidence rate curves, which suggests continuous community transmission in this area. Some studies reported that urban areas with a middle to low socioeconomic status are associated with higher numbers of COVID-19 cases and deaths^38–40^. An interesting signal is observed at the beginning of July 2021 in *Prinshoeveland* (Fig. 3). There is a considerable decrease followed by an increase in the local fractal dimension even though the corresponding incidence rate was low, particularly with sliding windows of 7 and 14 days. We assume that this increase might be useful as an early warning of increasing COVID-19 incidence in the coming period. Further analysis of this finding is required.

Based on the results from choropleth maps, some statistical sectors had similar, high fractal dimension estimates, despite having different incidence rate curves. This suggests that the complexity of COVID-19 incidence rates in these locations is relatively high and homogeneous. Studies in other fields also reported similar relationships between the fractal dimension and the complexity of the time series data^41–43^. However, we also need to take into account that the length of time series data plays an important role in calculating fractal dimensions. We could observe this effect since different lengths of the sliding window gave different fractal dimension estimates and thus different fractal dimension curves. The box-count estimator is particularly sensitive to the length of the data window and small amounts of data can have detrimental effects on its estimation, particularly when trends are presented^29,36^. This will eventually impact the ability to differentiate the clusters. Based on our simulation and real-world data, we recommend using the madogram or variogram estimators with shorter sliding windows, e.g., 7 or 14 days, in case of shorter time series data. With longer time series data, we could use the box-count or Hall-Wood estimator, but with longer sliding windows, e.g., 21 days, as well.

To our knowledge, the use of the fractal dimension combined with cluster detection is novel. The mean, variance, and autocorrelation values give a comprehensive summary of the COVID-19 daily incidence rate without having to explicitly examine individual daily incidence curves per statistical sector. This will be a major advantage when time is of the essence and one needs to evaluate multiple areas simultaneously. Again, we need to take into account that different fractal dimension estimators will give different results as shown in Web Figs. 11–18. In our example, since the box-count estimator gave a lower value of fractal dimension, it became difficult to classify areas into colors. The autocorrelation value given by the Hall-Wood estimator was very low to almost 0 while other methods gave relatively high values. Thus it is indeed important to compare different methods to obtain the best interpretation of the map. The simplified classification based on local fractal dimension could be useful to detect vulnerable areas. Local authorities could use this classification in their decision-making process related to targeted preventive measures.

Note that our proposed method has a different goal from scan-statistics tools, e.g. SaTScan, or model-based disease mapping methods. In essence, these methods investigate whether events of interest occur randomly. When this is not the case, they aim to find clusters based on anomalies in space and/or time. In contrast, our proposed method aims at capturing the complexity of a multivariate set of outcomes through time. We then classify these outcomes based on a set of characteristics of its complexity. In doing so, we explicitly avoid imposing a spatial mechanism as the data-generating process, since we want to obtain insight in (dis-)similarities in the complexity, regardless of their geographical location.

Some limitations have to be mentioned. First, we used retrospective data from specific locations and periods. This means that the findings are difficult to generalize to other settings. However, this specificity might help local authorities to describe the situation and make decisions related to mitigation strategies. Second, we did not set a threshold to flag the local fractal dimension, that is, when it is high enough to warrant a warning or flagging. Based on multidisciplinary input, this would be needed when the fractal dimension would be used as a component of an early warning system.

## Conclusion

In conclusion, the fractal perspective of time series analysis offers useful insight into the evolution of an epidemic curve. The choice of the fractal dimension estimator and related parameters should be considered carefully when selecting the appropriate method to use. Fractal dimension analysis may also provide further insight into the wide heterogeneity in the transmission of the virus due to large differences between individuals in infectiousness, susceptibility, and contact behavior.

## Supplementary Information

Supplementary Information 1.

Supplementary Information 2.

Supplementary Information 3.

Supplementary Information 4.

Supplementary Information 5.

Supplementary Information 6.

Supplementary Information 7.

Supplementary Information 8.

Supplementary Information 9.

Supplementary Information 10.

Supplementary Information 11.

Supplementary Information 12.

Supplementary Information 13.

Supplementary Information 14.

Supplementary Information 15.

Supplementary Information 16.

Supplementary Information 17.

Supplementary Information 18.

Supplementary Information 19.

## Data Availability

R code for simulation study is available from https://github.com/yessikanatalia/fractdim_sim.git. The daily COVID-19 data that support the findings of this study are available from the Agency for Care and Health but restrictions apply to the availability of these data, which were used under license for the current study, and so are not publicly available. Data are however available from author N.H. upon reasonable request and with permission of the Agency for Care and Health.
