# Supplementary figures and images for: Fractal dimension based geographical clustering of COVID-19 time series data
Source: Sci Rep. 2023 Mar 15;13:4322. doi: 10.1038/s41598-023-30948-7 (PMC10016183; doi:10.1038/s41598-023-30948-7)

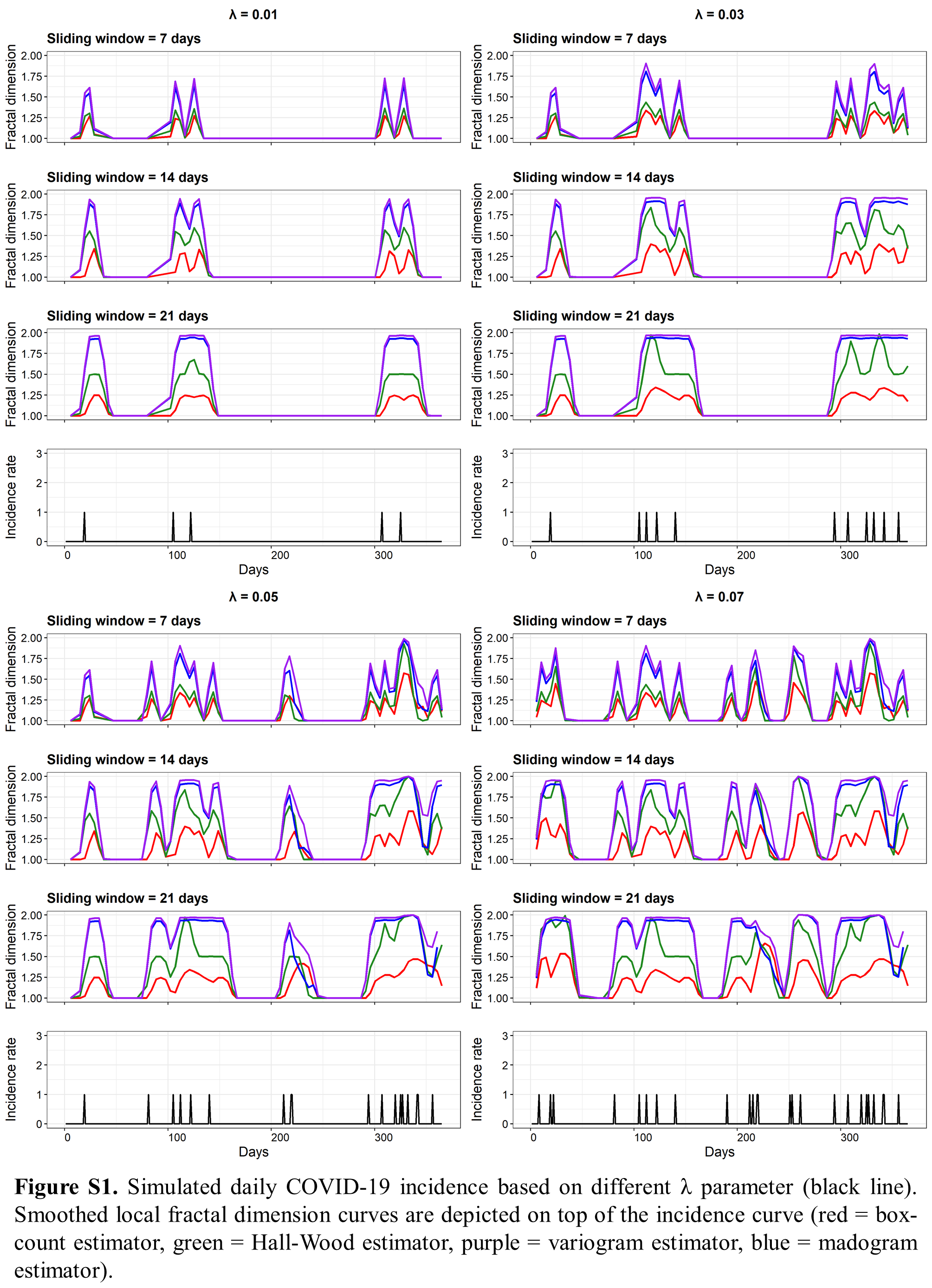

Supplement: Supplementary file 1 — Supplementary Information 1. [file 41598_2023_30948_MOESM1_ESM.tif]

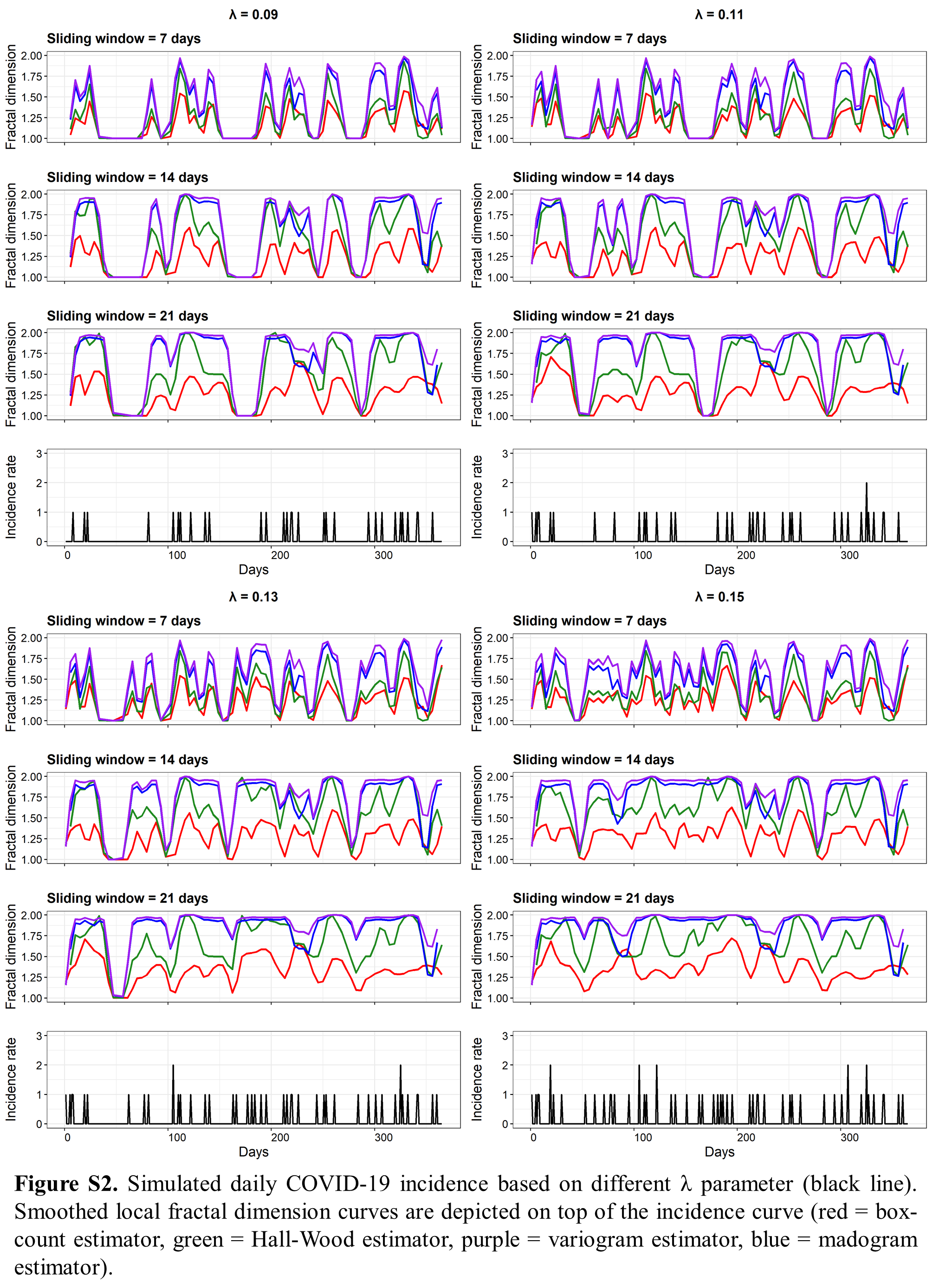

Supplement: Supplementary file 2 — Supplementary Information 2. [file 41598_2023_30948_MOESM2_ESM.tif]

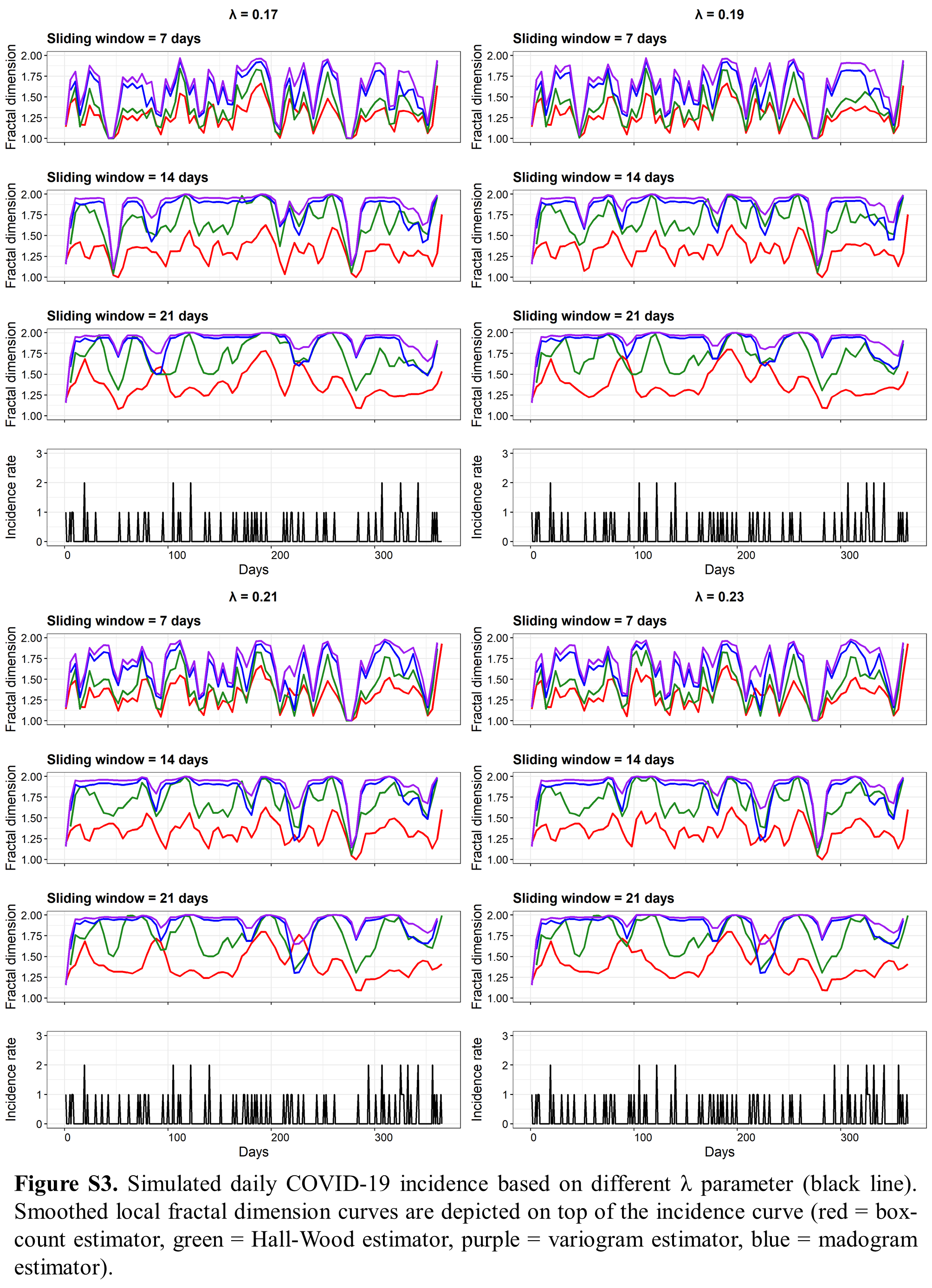

Supplement: Supplementary file 3 — Supplementary Information 3. [file 41598_2023_30948_MOESM3_ESM.tif]

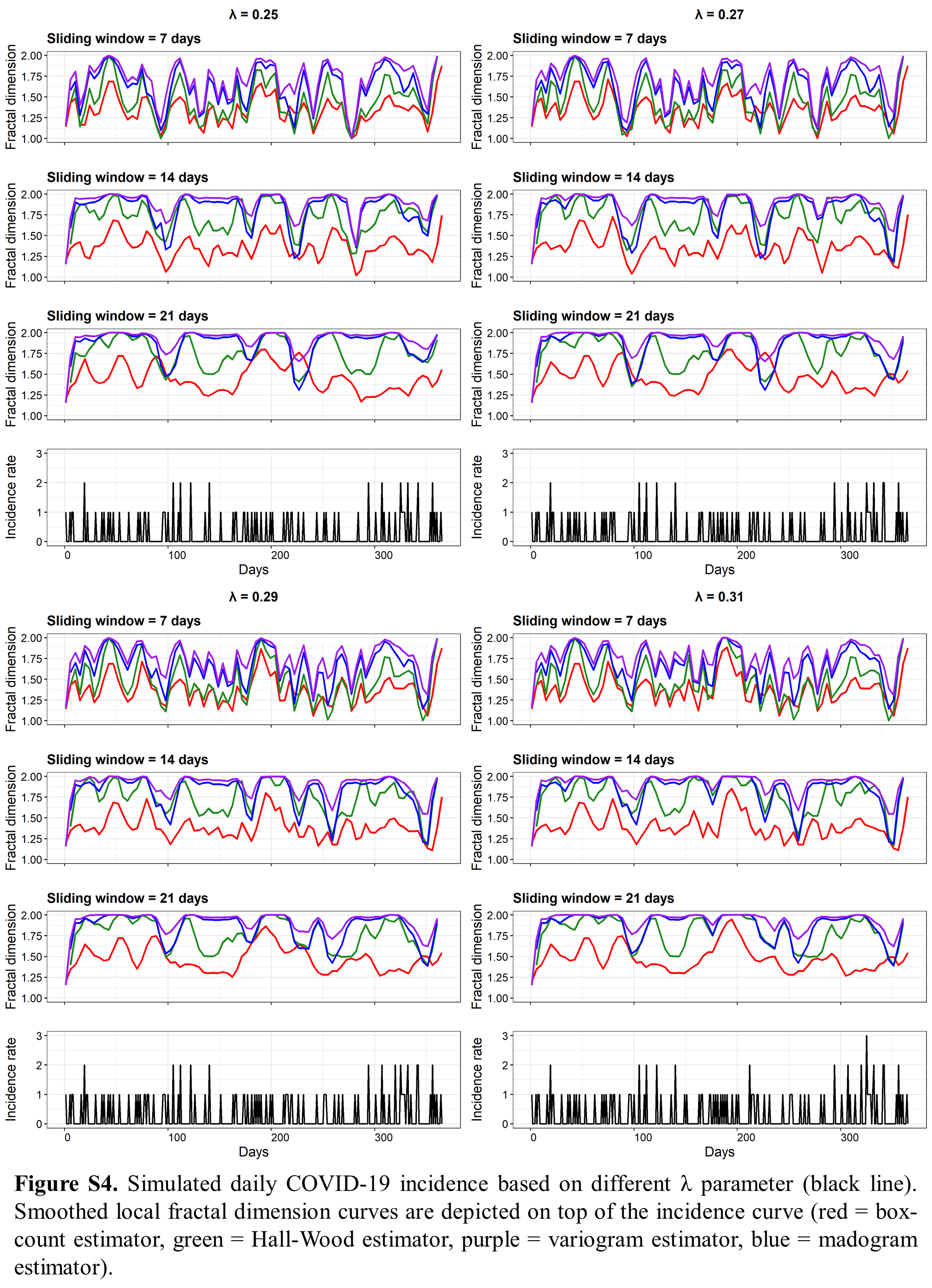

Supplement: Supplementary file 4 — Supplementary Information 4. [file 41598_2023_30948_MOESM4_ESM.tif]

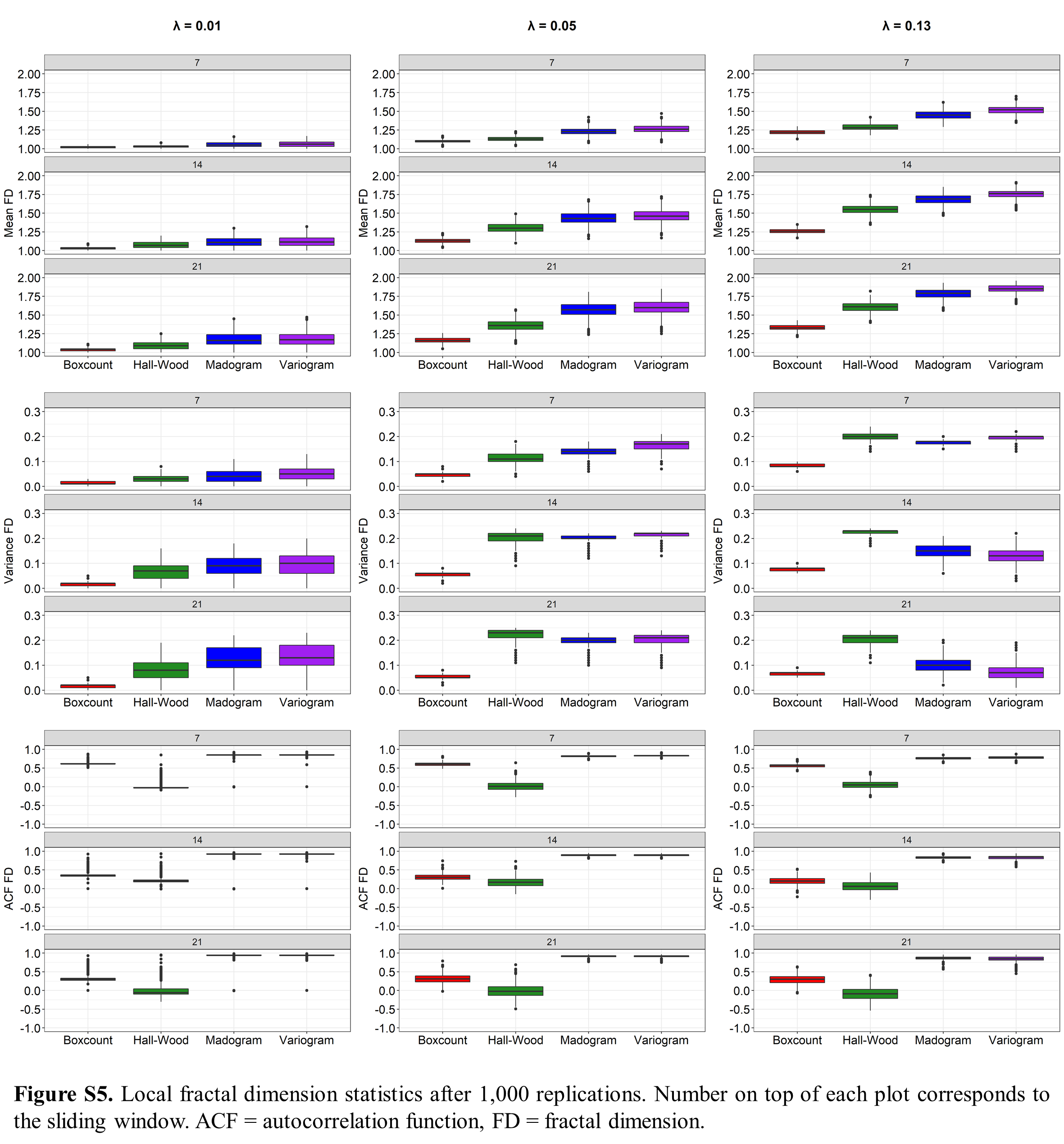

Supplement: Supplementary file 5 — Supplementary Information 5. [file 41598_2023_30948_MOESM5_ESM.tif]

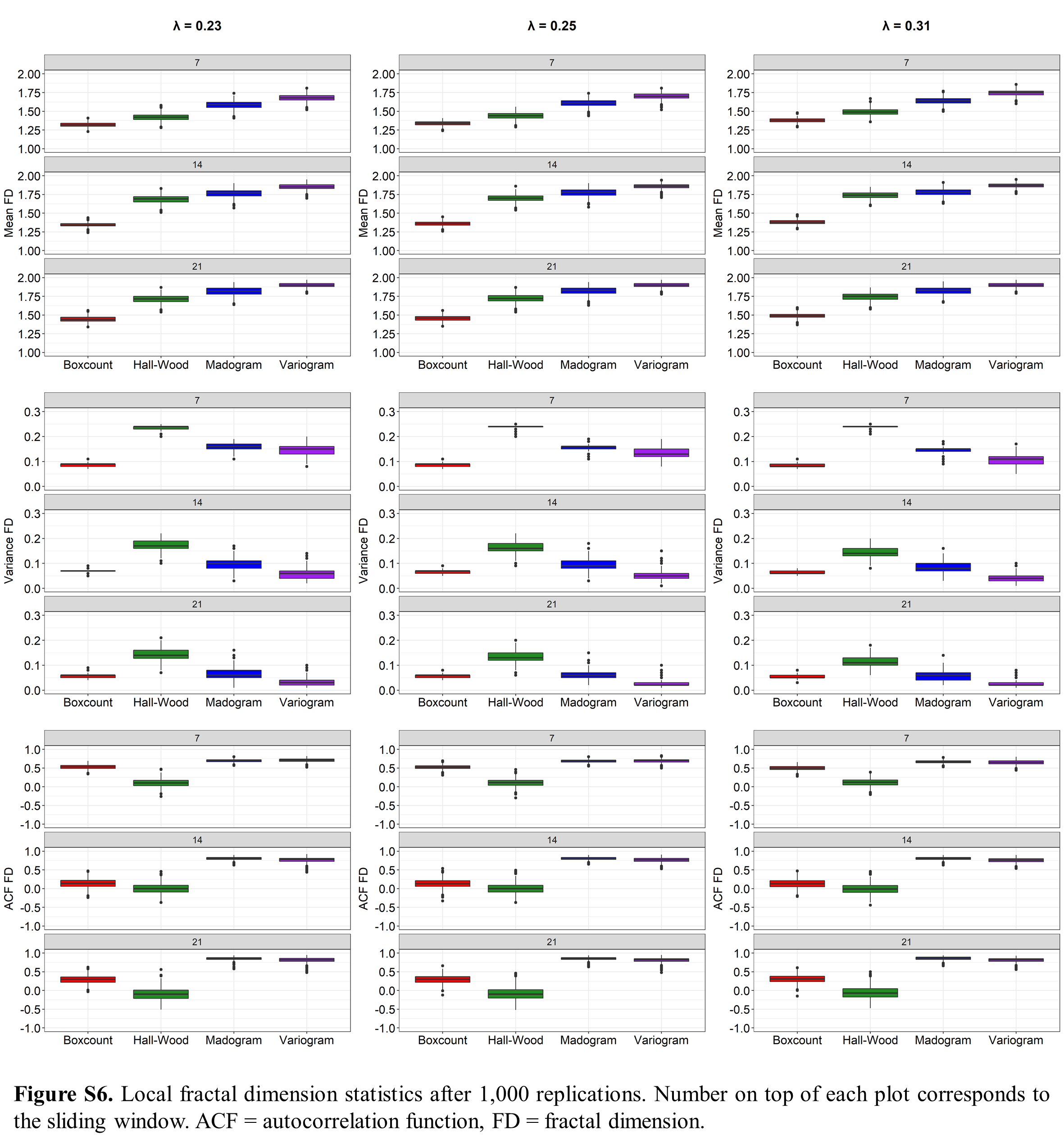

Supplement: Supplementary file 6 — Supplementary Information 6. [file 41598_2023_30948_MOESM6_ESM.tif]

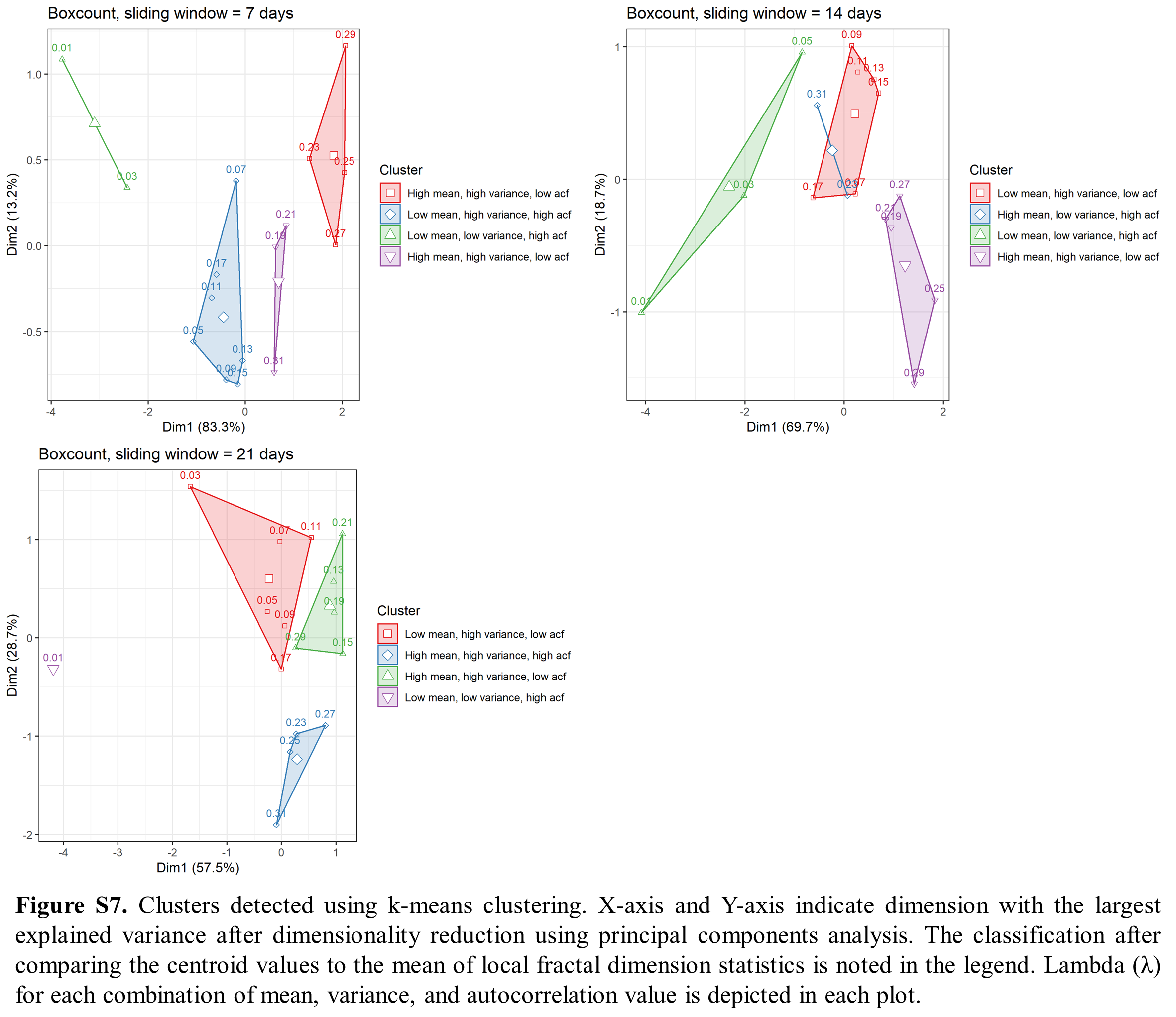

Supplement: Supplementary file 7 — Supplementary Information 7. [file 41598_2023_30948_MOESM7_ESM.tif]

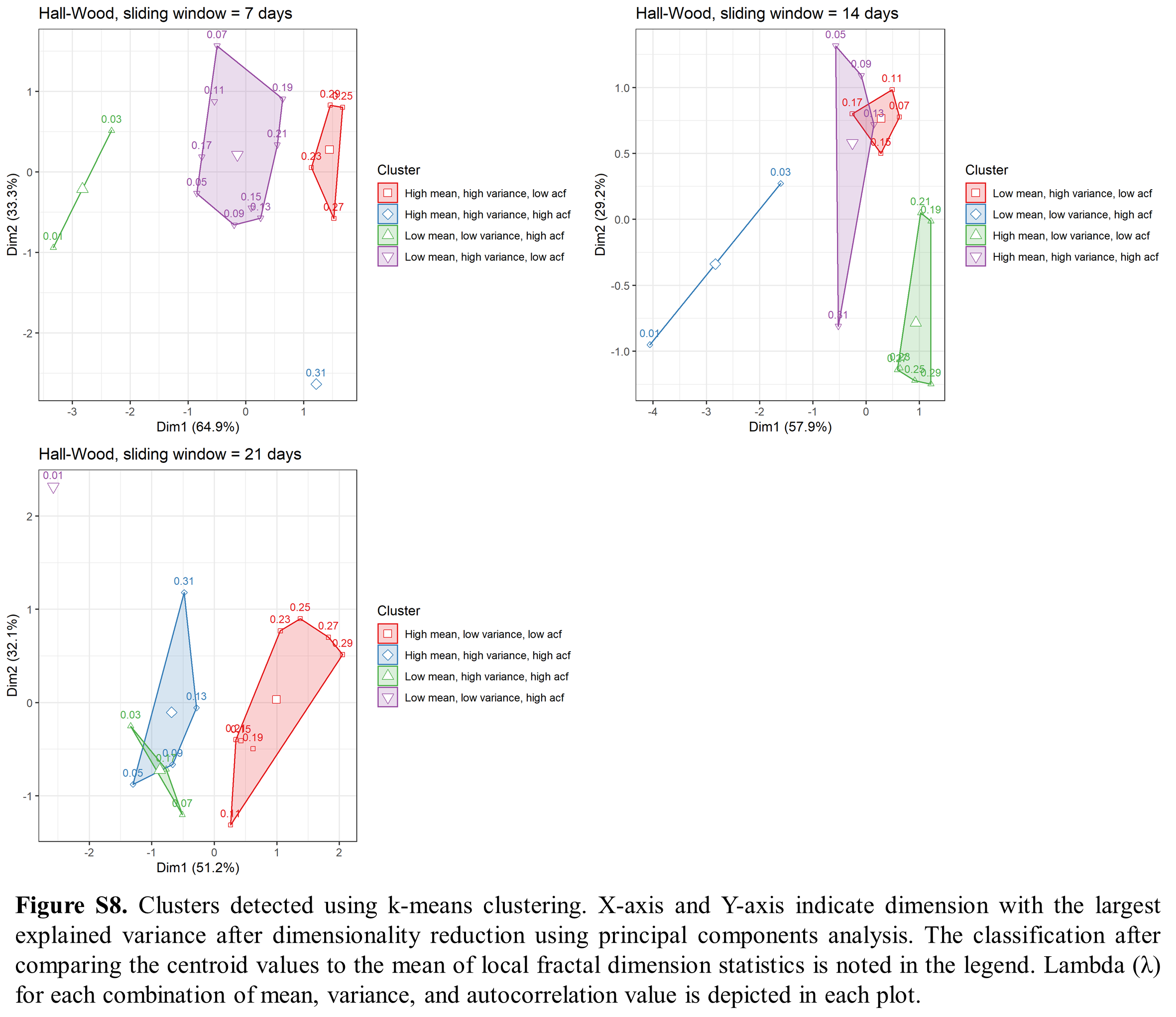

Supplement: Supplementary file 8 — Supplementary Information 8. [file 41598_2023_30948_MOESM8_ESM.tif]

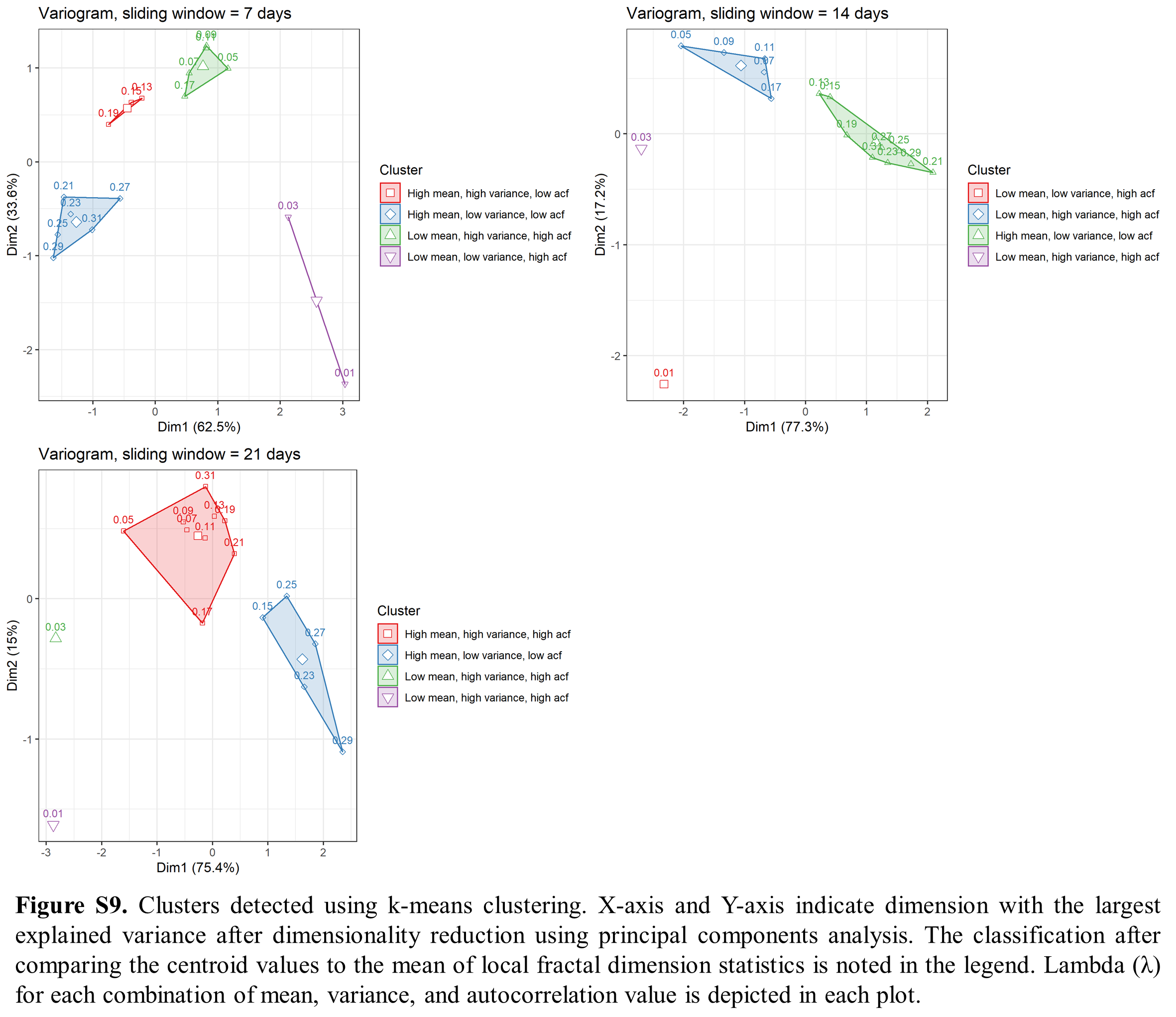

Supplement: Supplementary file 9 — Supplementary Information 9. [file 41598_2023_30948_MOESM9_ESM.tif]

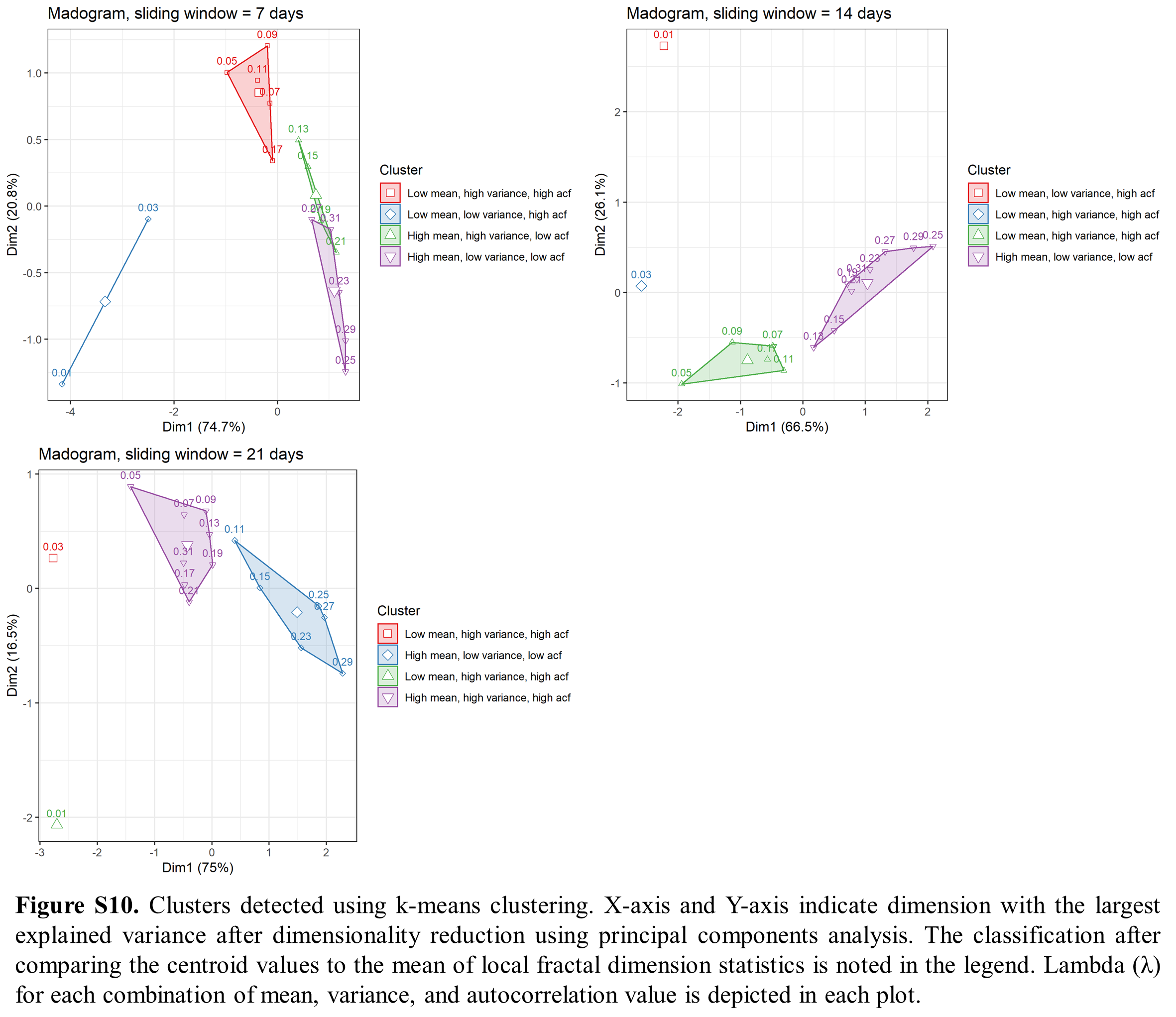

Supplement: Supplementary file 10 — Supplementary Information 10. [file 41598_2023_30948_MOESM10_ESM.tif]

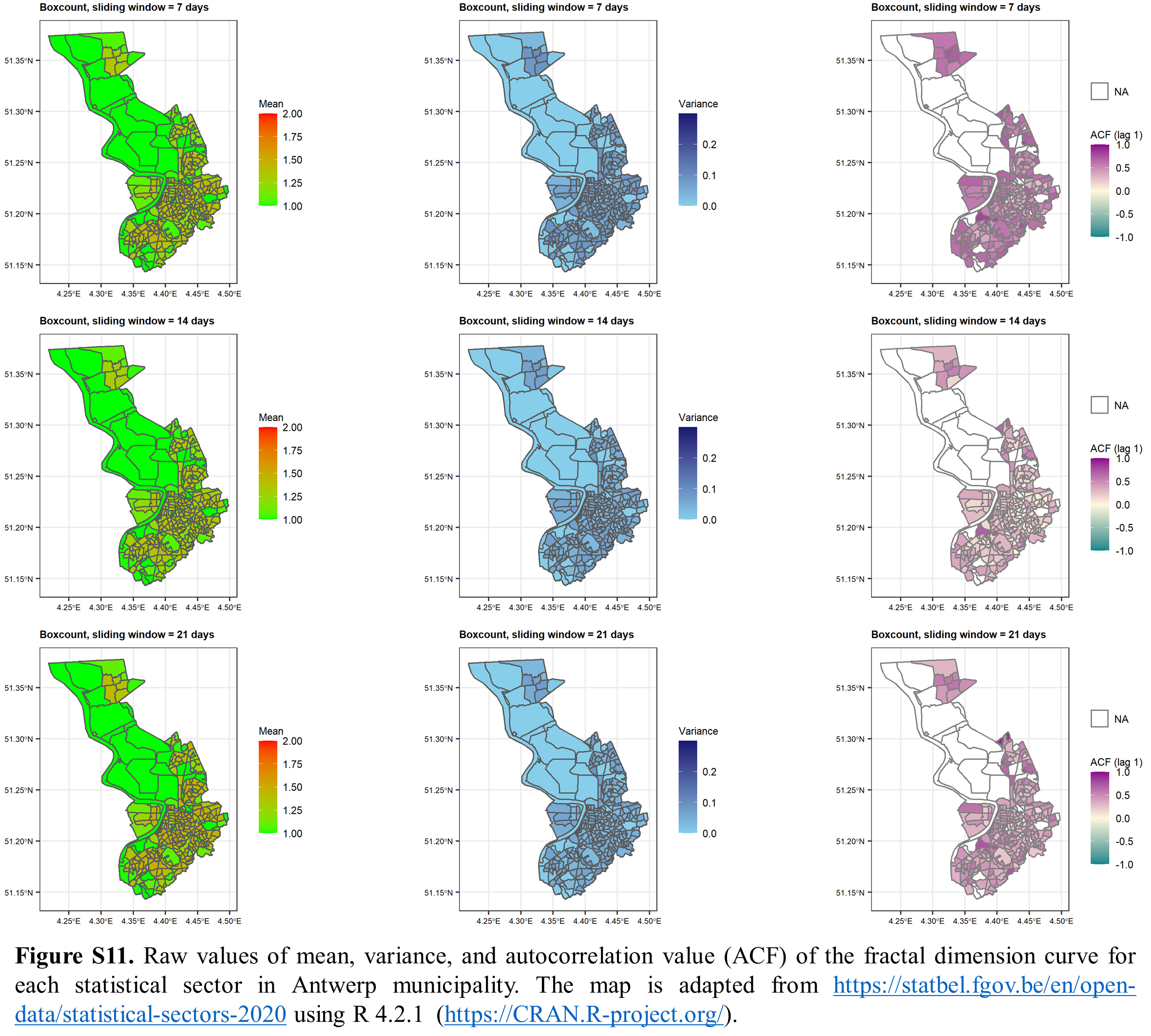

Supplement: Supplementary file 11 — Supplementary Information 11. [file 41598_2023_30948_MOESM11_ESM.tif]

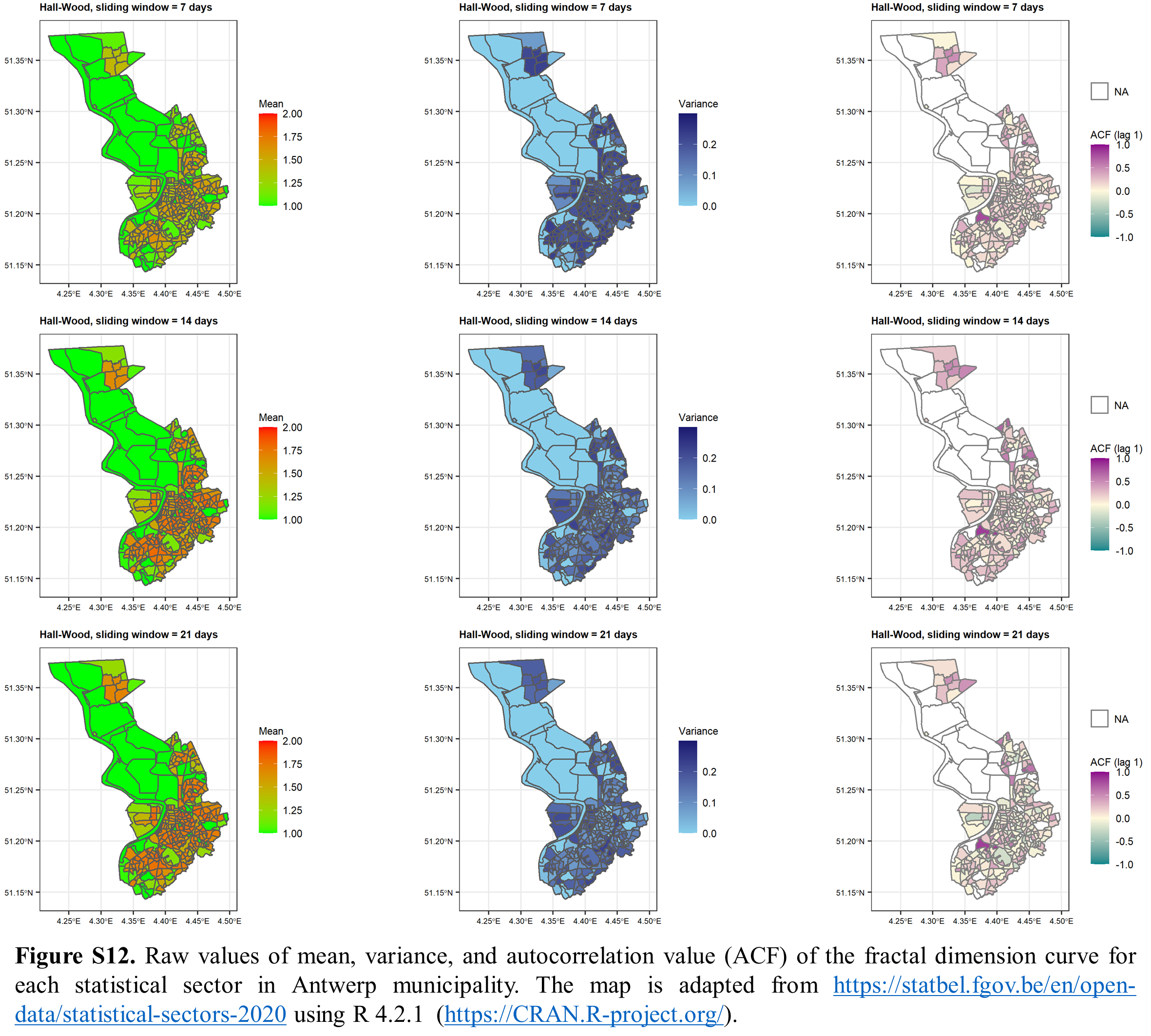

Supplement: Supplementary file 12 — Supplementary Information 12. [file 41598_2023_30948_MOESM12_ESM.tif]

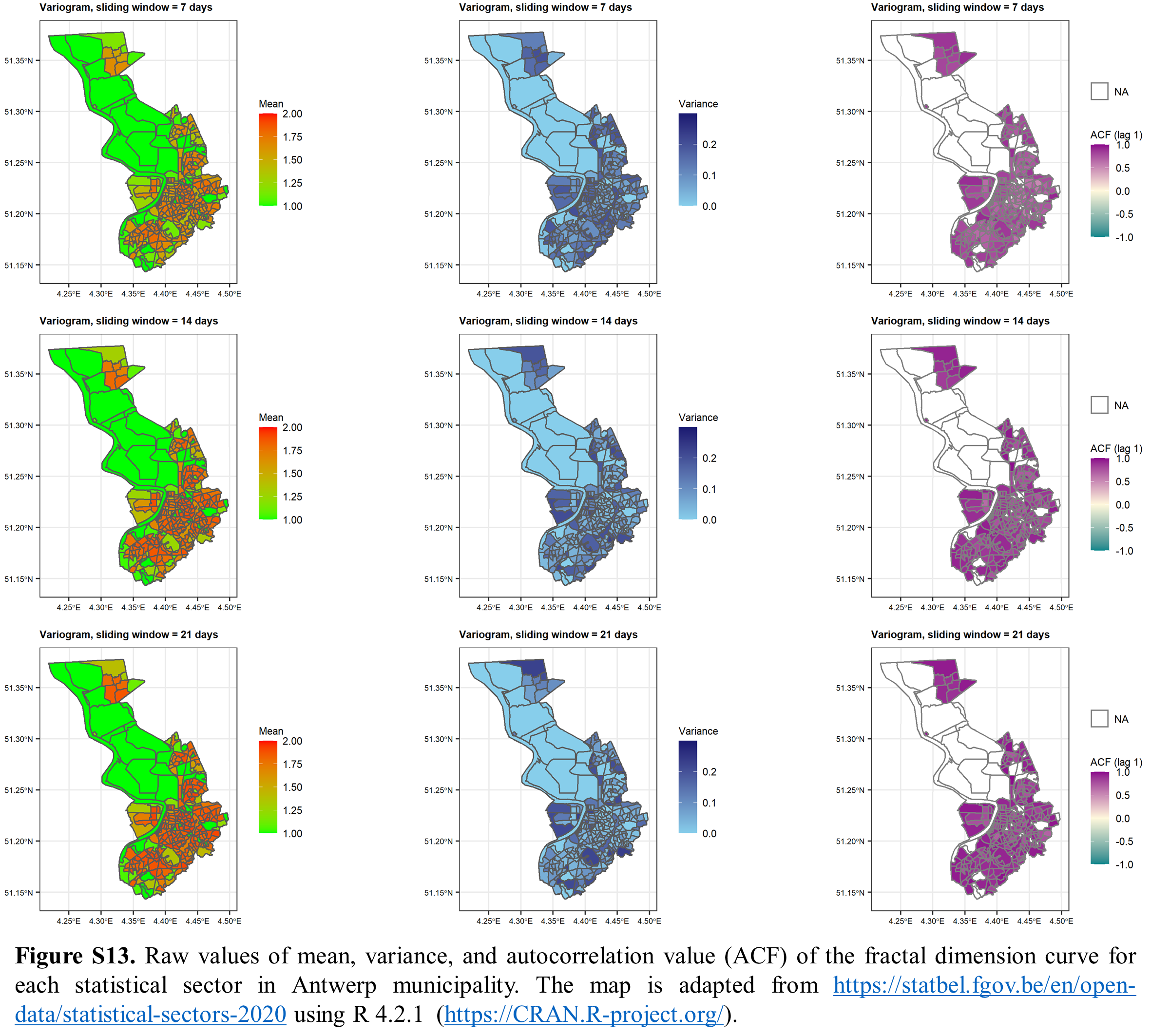

Supplement: Supplementary file 13 — Supplementary Information 13. [file 41598_2023_30948_MOESM13_ESM.tif]

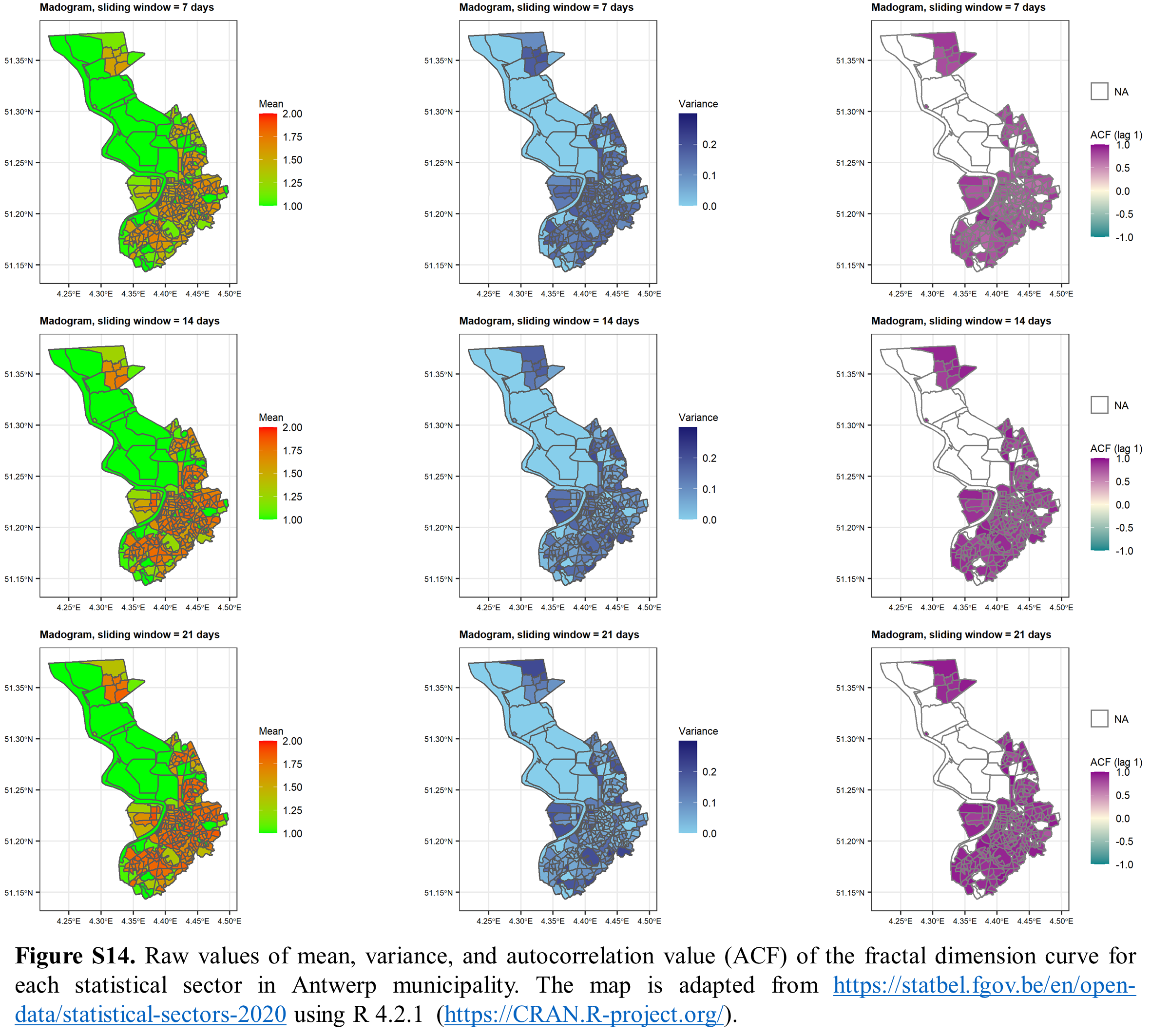

Supplement: Supplementary file 14 — Supplementary Information 14. [file 41598_2023_30948_MOESM14_ESM.tif]

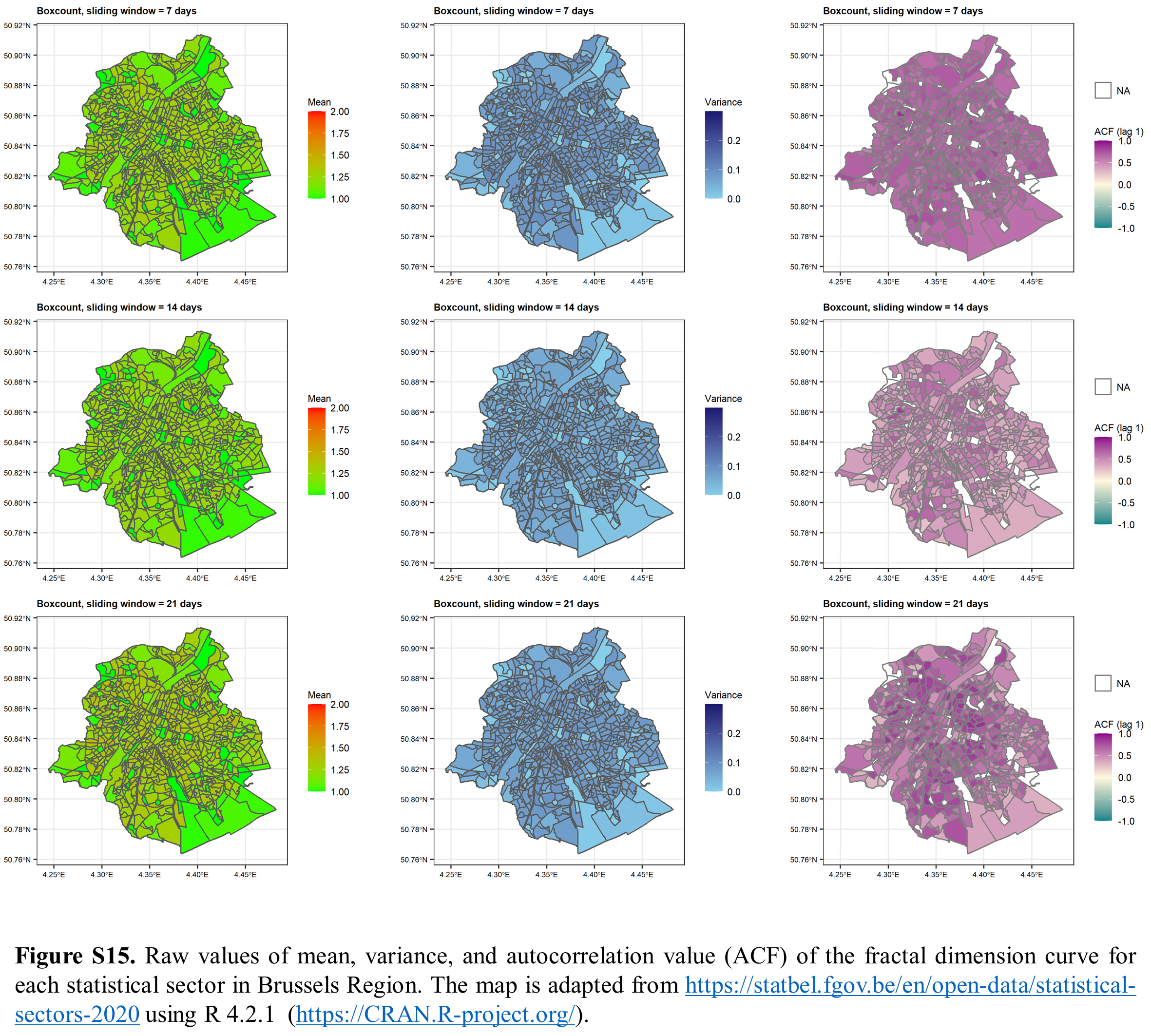

Supplement: Supplementary file 15 — Supplementary Information 15. [file 41598_2023_30948_MOESM15_ESM.tif]

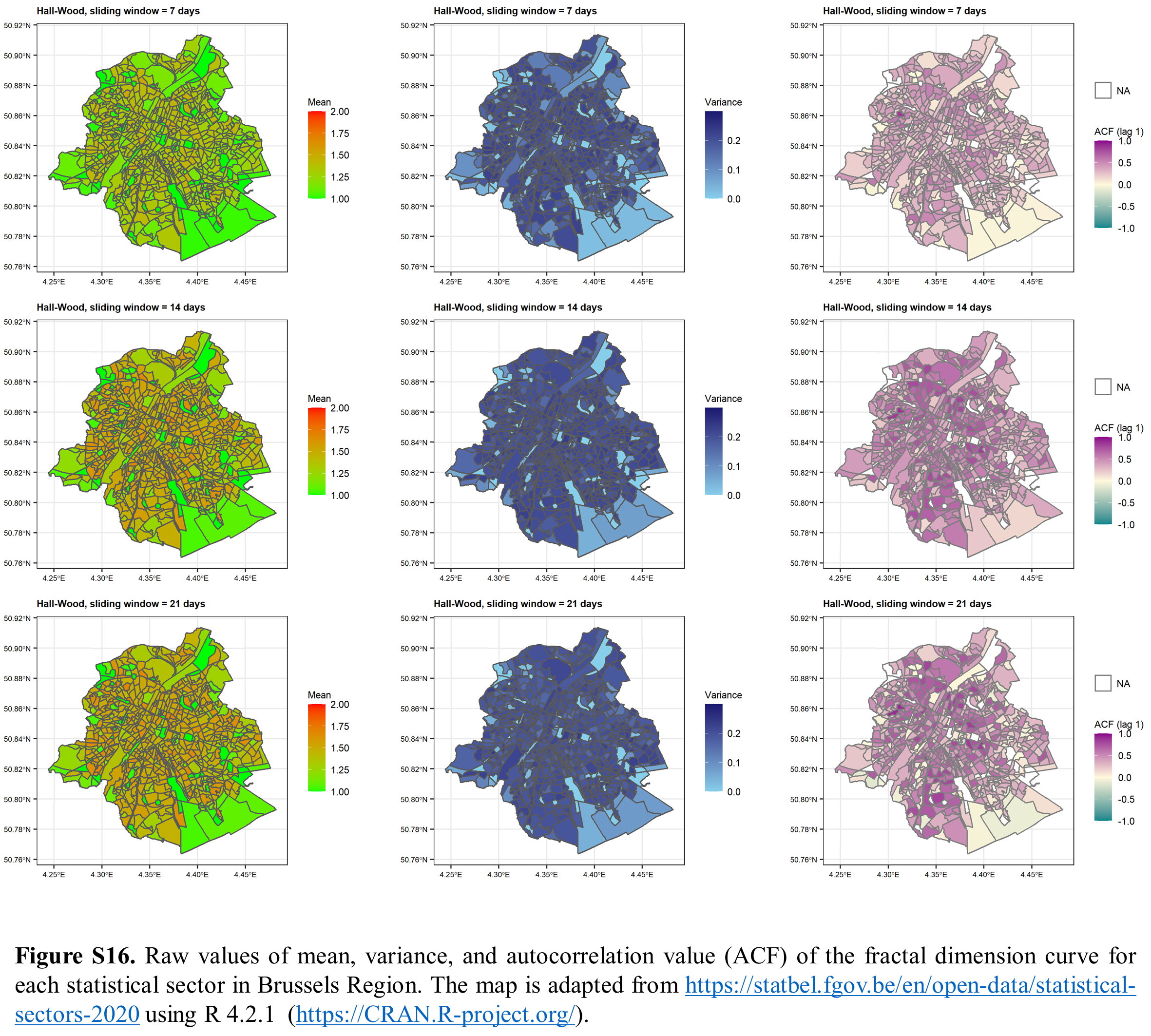

Supplement: Supplementary file 16 — Supplementary Information 16. [file 41598_2023_30948_MOESM16_ESM.tif]

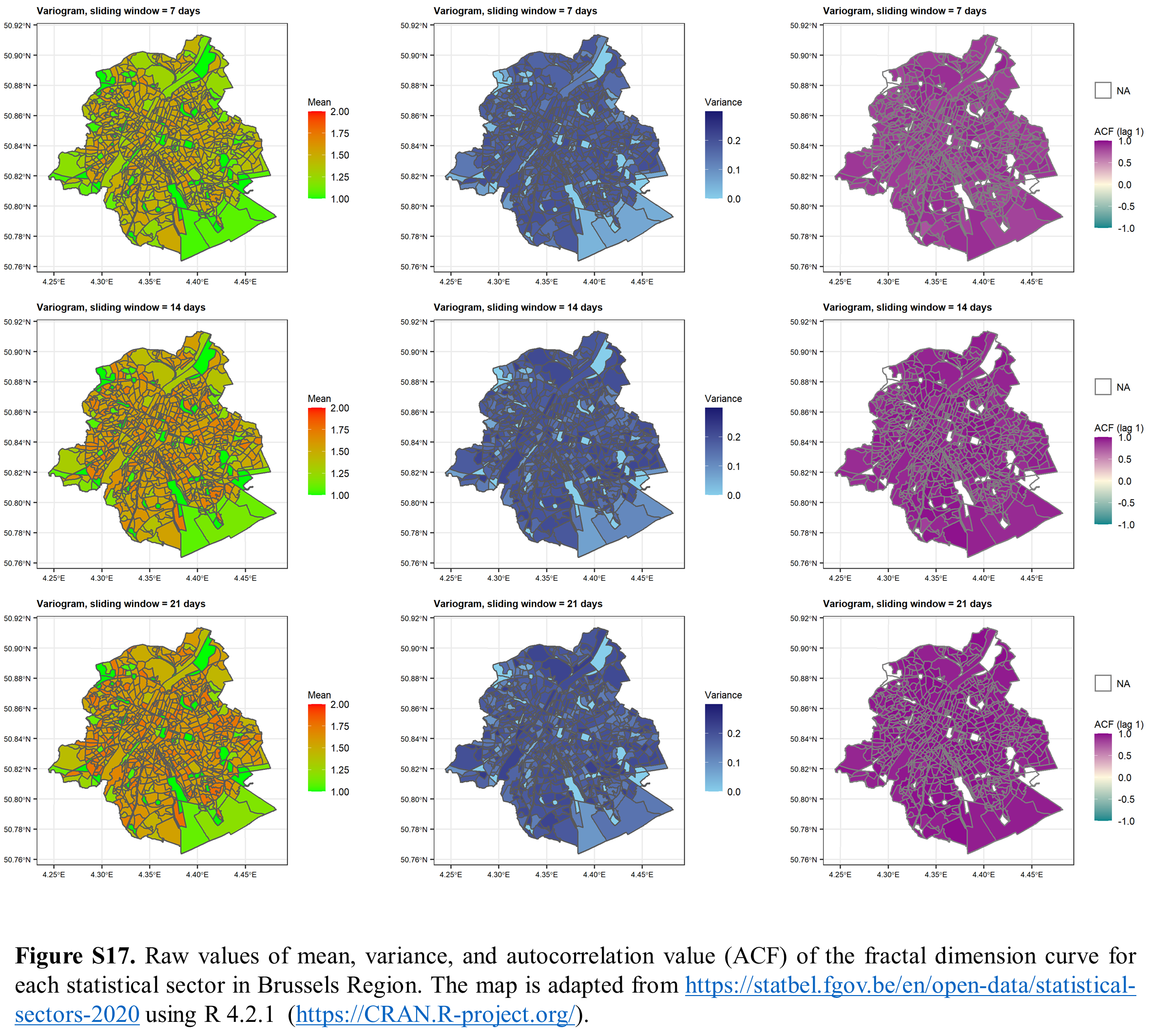

Supplement: Supplementary file 17 — Supplementary Information 17. [file 41598_2023_30948_MOESM17_ESM.tif]

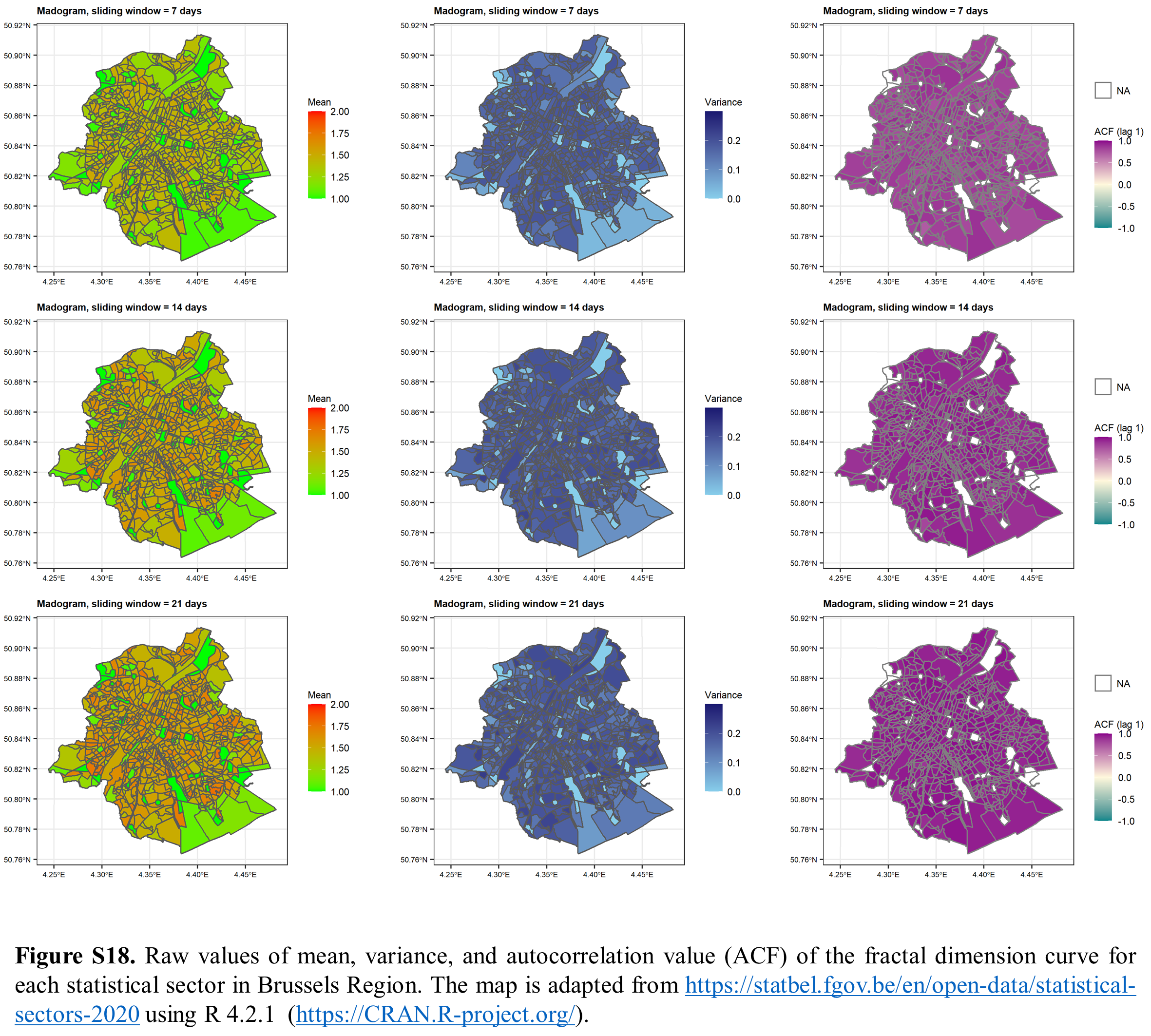

Supplement: Supplementary file 18 — Supplementary Information 18. [file 41598_2023_30948_MOESM18_ESM.tif]

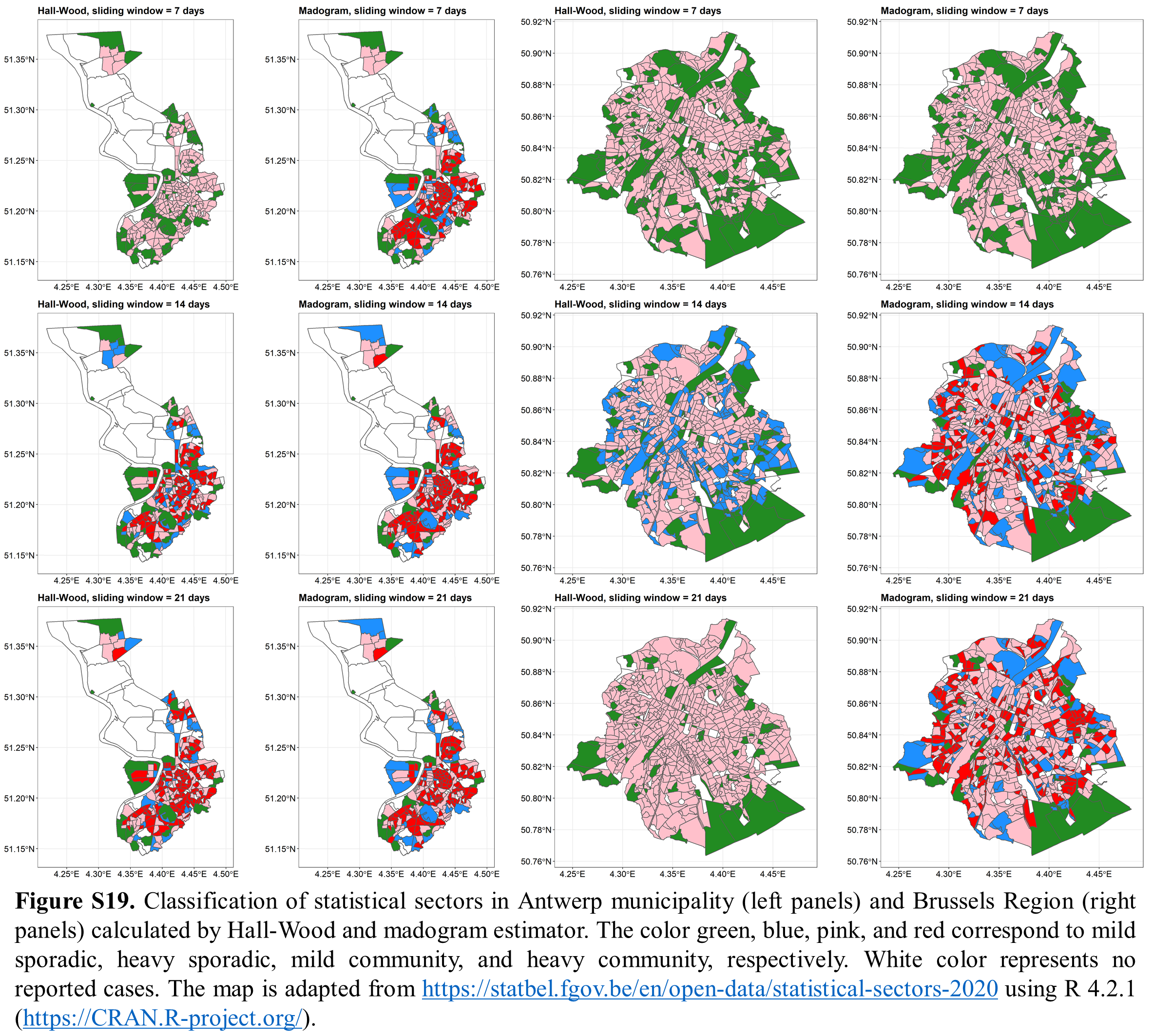

Supplement: Supplementary file 19 — Supplementary Information 19. [file 41598_2023_30948_MOESM19_ESM.tif]
